# Secretome from estrogen-responding human placenta-derived mesenchymal stem cells rescues ovarian function and circadian rhythm in mice with cyclophosphamide-induced primary ovarian insufficiency

**DOI:** 10.1186/s12929-024-01085-8

**Published:** 2024-10-11

**Authors:** Duy-Cuong Le, Mai-Huong T. Ngo, Yung-Che Kuo, Shu-Hwa Chen, Chung-Yen Lin, Thai-Yen Ling, Quoc Thao Trang Pham, Heng-Kien Au, Jihwan Myung, Yen-Hua Huang

**Affiliations:** 1 International Ph.D. Program in Cell Therapy and Regenerative Medicine, College of Medicine, Taipei Medical University, Taipei, 11031 Taiwan; 2 Laboratory, Vinmec International Hospital, Minh Khai Street, Hai Ba Trung, Hanoi, Vietnam; 3 Department of Biochemistry and Molecular Cell Biology, School of Medicine, College of Medicine, Taipei Medical University, 250 Wuxing Street, Taipei, 11031 Taiwan; 4 TMU Research Center for Cell Therapy and Regeneration Medicine, Taipei Medical University, 250 Wuxing Street, Taipei, 11031 Taiwan; 5 TMU Research Center of Cancer Translational Medicine, Taipei Medical University, Taipei, 11031 Taiwan; 6 Institute of Information Science, Academia Sinica, Taipei, 11529 Taiwan; 7 Institute of Fishery Sciences, College of Life Science, National Taiwan University, Taipei, 10617 Taiwan; 8 Genome and Systems Biology Degree Program, National Taiwan University, Taipei, 10617 Taiwan; 9 Department and Graduate Institute of Pharmacology, College of Medicine, National Taiwan University, Taipei, 10617 Taiwan; 10 Department of Obstetrics and Gynecology, School of Medicine, College of Medicine, Taipei Medical University, 250 Wuxing Street, Taipei, 11031 Taiwan; 11 Department of Obstetrics and Gynecology, Taipei Medical University Hospital, Taipei, 11042 Taiwan; 12 Center for Reproductive Medicine, Taipei Medical University Hospital, Taipei Medical University, Taipei, 11042 Taiwan; 13 Graduate Institute of Mind, Brain and Consciousness, Taipei Medical University, 250 Wuxing Street, Taipei, 11031 Taiwan; 14 Brain and Consciousness Research Centre (BCRC), TMU-Shuang Ho Hospital, New Taipei City, 23561 Taiwan; 15 Graduate Institute of Medical Sciences, College of Medicine, Taipei Medical University, Wuxing Street, Taipei, 11031 Taiwan

**Keywords:** Placenta mesenchymal stem cell, Estrogen receptor, Primary ovarian insufficiency, Ovarian circadian rhythm disorder, Angiogenin, Exosomal miRNA

## Abstract

**Background:**

Primary ovarian insufficiency (POI) is an early decline in ovarian function that leads to ovarian failure. Conventional treatments for POI are inadequate, and treatments based on mesenchymal stem cells (MSCs) have emerged as an option. However, the lack of consideration of the estrogen niche in ovarian tissue significantly reduces the therapeutic efficacy, with an unclear mechanism in the MSCs in POI treatment. Furthermore, the disruption of circadian rhythm associated with POI has not been previously addressed.

**Methods:**

Conditioned medium (CM) and estradiol-conditioned medium (E2-CM) were generated from estrogen receptor positive MSCs (ER^+^pcMSCs). Chemotherapy-induced POI models were established using C57BL/6 mice (in vivo) and KGN cells (in vitro) treated with cyclophosphamide (CTX) or 4-hydroperoxycyclophosphamide (4-OOH-CP). Gene/protein expressions were detected using RT-qPCR, Western blotting, and immunohistochemistry assays. Locomotor activity was monitored for behavioral circadian rhythmicity. Cytokine arrays and miRNA analysis were conducted to analyze potential factors within CM/E2-CM.

**Results:**

The secretome of ER^+^pcMSCs (CM and E2-CM) significantly reduced the CTX-induced defects in ovarian folliculogenesis and circadian rhythm. CM/E2-CM also reduced granulosa cell apoptosis and rescued angiogenesis in POI ovarian tissues. E2-CM had a more favorable effect than the CM. Notably, ER^+^pcMSC secretome restored CTX-induced circadian rhythm defects, including the gene expressions associated with the ovarian circadian clock (e.g., *Rora, E4bp4, Rev-erbα*, *Per2* and *Dbp*) and locomotor activity. Additionally, the cytokine array analysis revealed a significant increase in cytokines and growth factors associated with immunomodulation and angiogenesis, including angiogenin. Neutralizing the angiogenin in CM/E2-CM significantly reduced its ability to promote HUVEC tube formation in vitro. Exosomal miRNA analysis revealed the miRNAs involved in targeting the genes associated with POI rescue (*PTEN* and *PDCD4*)*,* apoptosis (caspase-3, BIM), estrogen synthesis (*CYP19A1*), ovarian clock regulation (*E4BP4*, *REV-ERBα*) and fibrosis (*COL1A1*).

**Conclusion:**

This study is the first to demonstrate that, in considering the estrogen niche in ovarian tissue, an estrogen-priming ER^+^pcMSC secretome achieved ovarian regeneration and restored the circadian rhythm in a CTX-induced POI mouse model. The potential factors involved include angiogenin and exosomal miRNAs in the ER^+^pcMSC secretome. These findings offer insights into potential stem cell therapies for chemotherapy-induced POI and circadian rhythm disruption.

**Supplementary Information:**

The online version contains supplementary material available at 10.1186/s12929-024-01085-8.

## Introduction

Primary ovarian insufficiency (POI), also referred to as premature ovarian failure (POF), is characterized by an irreversible decline in ovarian function and estrogen levels in women younger than 40 years [1]. The prevalence is high and varies by ethnicity, and it causes infertility in approximately 1% of women of reproductive age [1, 2]. The goals of POI treatment are to restore hormonal balance and reproductive function. However, the available treatment strategies for POI exhibit low efficacy [2].

The typical manifestations of a decrease or loss of folliculogenesis are an increase in follicle-stimulating hormone (FSH) levels (to menopausal levels [> 40 mIU/ml]) [3, 4]; hypoestrogenism [5, 6]; low anti-Müllerian hormone (AMH) levels; irregular menstruation; and ultimately, amenorrhea. POI is diagnosed on the basis of depleted ovarian reserves at a young age [7, 8], and idiopathic POI is the most common etiology (80–90%). POI is also caused by genetic and autoimmune diseases, radiation, increased reactive oxygen species (ROS) levels, and chemotherapy which can induce DNA damage leading to increased follicle cell apoptosis and atresia, and impaired folliculogenesis [4, 9]. After undergoing chemotherapy, 34% of women with initially normal ovarian function developed POI [10].

Chemotherapy is a major contributor to POI development. Chemotherapy-induced DNA damage, such as DNA double-stranded breaks, can induce the apoptosis of granulosa cells and oocytes in the ovaries [11]. These effects occur through two mechanisms, namely phosphatidylinositol 3-kinase (PI3K) dependent primordial follicle activation and tumor suppressor protein TP53–dependent apoptosis [12]. Chemotherapeutic drugs activate the PI3K/Akt signaling pathway, causing continuous activation of dormant primordial follicles [13] and failure of the dormant follicle pool [11]. Chemotherapy-induced ovarian damage may also involve vascular damage and ovarian cortex fibrosis [14].

Cyclophosphamide (CTX) is widely used in cancer treatment because its metabolite, phosphoramide mustard, exhibits anticancer properties [15]. In the ovaries, CTX metabolites cause crosslinking of oocyte DNA, inhibiting their synthesis and function, and therefore, CTX was used to induce POI in mouse models [16]. Although systemic hormone replacement therapy is the treatment of choice for alleviating menopausal symptoms in patients with POI, it exhibits unsatisfactory efficacy in improving reproductive function. Moreover, long-term replacement with exogenous hormones can increase the risk of breast and endometrial cancer [5]. Other treatment strategies include ovarian tissue transplantation and oocyte donation. However, these strategies are not widely accepted [17]. Oocyte freezing before chemotherapy, which involves surgical oocyte pickup, was also proposed as a strategy [18].

Estrogen was demonstrated to regulate the peripheral tissue clock and metabolic rhythm [19]. A decrease in ovarian reserve is believed to be related to changes in the ovarian circadian rhythm [20]. A bi-directional connection can exist between the suprachiasmatic nucleus (SCN) and the ovaries. The ovarian circadian rhythm is synchronized with the SCN through gonadotropins [21]. Meanwhile, circulating estradiol has been reported to influence the SCN through estrogen receptors (ER) [22, 23]. For example, the expression of clock genes such as *Bmal1, Clock, Per1,* and *Per2* is regulated by estrogen through estrogen response elements (EREs), and the expression of ER is directly controlled by BMAL1 and CLOCK through the E-box on ERα and ERβ [19]. Furthermore, a reciprocal relationship exists between estrogen and the circadian rhythm. For example, estrogen-mediated CLOCK sumoylation upregulates the transcriptional activity of ERα [24], and estrogen-induced PER2 binding enhances ERα degradation; by contrast, suppression of Per2 levels leads to ERα stabilization [25]. Additionally, impaired fertility has been observed in mutant mice with circadian clock disorder, and both complete and ovarian conditional knockout of *Bmal1* have been reported to result in implantation failure in female mice [26, 27].

Clinical trials have utilized stem cell therapy, particularly mesenchymal stem cell (MSC) therapy, to treat patients with POI. To date, 29 clinical trials have reported on the use of autologous or allogeneic MSCs for POI treatment (https://clinicaltrials.gov/). Additionally, cellular derivatives of MSCs, such as MSC-conditioned medium (MSC-CM), which contains paracrine factors, and extracellular vesicles, including exosomes, have also been used to treat POI [28–31]. However, the MSCs used in current clinical trials are derived from bone marrow, adipose tissues, or umbilical cord, which cannot respond to estrogen in the ovarian niche due to the absence of estrogen receptors (ERs) (Supplemental Fig. S2, [32–35]). Thus far, no clinical trial has tested the use of MSC-CM (secretome) to treat POI. Notably, the effects of precision therapy involving the MSC secretome on mediating ovarian regeneration and restoring the circadian rhythm, particularly in response to estrogen in the ovarian niche, remain unclear.

In the present study, considering the estrogen niche in ovarian tissues, we identified MSCs from human placenta choriodecidual membrane tissues that express ERs (ER^+^pcMSCs) as ideal candidates for utilizing the MSC secretome in the treatment of POI. We evaluated the potential therapeutic effects of the ER^+^pcMSC secretome, with or without estradiol (E2) priming (ER^+^pcMSC-CM/E2-CM), on CTX-induced POI and circadian rhythm regulation, both in vitro and in vivo. The underlying mechanisms were further investigated through cytokine arrays and exosomal miRNA analysis. This study is the first to consider the estrogen niche in ovarian tissues and demonstrates the potential therapeutic effect of the ER^+^pcMSC secretome on chemotherapy-induced POI through the promotion of ovarian regeneration and the restoration of diurnal rhythm.

## Materials and methods

### Generation of ER^+^pcMSCs

In the present study, human placentas were donated by women who received cesarean sections in Taipei Medical University Hospital with procedures approved by the Institutional Review Board at Taipei Medical University (IRB Approval No. TMU-JIRB 201501063 and TMU-JIRB N202304143). Written informed consent was obtained from all donors and experiments were performed in accordance with relevant guidelines and regulations.

The pcMSCs from human placental tissues (provided by Prof. Thai-Yen Ling’s Lab in National Taiwan University) were isolated and characterized as described in our previous study [36]. In brief, the placental tissues were carefully dissected, and the harvested tissue pieces were washed several times with phosphate-buffered saline (PBS); minced; and enzymatically digested using digestion buffer containing DNase I, protease, and collagenase B in minimum essential medium (MEM, Thermo Fisher Scientific, NY, USA). The digested tissues were neutralized with 10% fetal bovine serum (FBS) in MCDB201 medium (Sigma-Aldrich, MO, USA) and filtered twice through a nylon membrane (pore size: 100 μm) and, subsequently a 100 μm cell strainer (BD Bioscience, NJ, USA) to remove undigested pieces. The mixture was centrifuged at 300 × g for 20 min, after which the supernatant was discarded. The cell pellets were resuspended and cultured in ITS and 10 mg/ml EGF in MCDB201 at 37°C and 5% CO_2_. The pcMSCs from passage 6 were subjected to phenotypic marker identification through flow cytometry to identify their MSC characteristics [36].

### Generation of CM and E2-CM derived from ER^+^pcMSCs

To investigate the differences in protein concentrations between CM and E2-CM, pcMSCs were cultured in serum-free MCDB201 medium; for this procedure the seeding number (5 × 10^5^ cells/10 cm dish) and harvest duration was set at 48 h. At approximately 50% confluence, the medium was replaced with fresh medium containing E2 (100 nM) for 48 h. The culture dishes were washed three times with PBS, and serum-free medium was added. The cells were cultured for another 48 h to obtain E2-CM. Normal CM was also harvested by using this procedure without the E2 priming. CM and E2-CM were then subsequently concentrated 10- or 50-fold by using an ultraconcentrator (Vivaspin 20 with 3000 Dalton molecular weight cutoff filters, Cytiva, USA) per the manufacturer’s instructions and were stored at − 80°C for future use.

### Human granulosa-like cell line (KGN)

Cells from the human granulosa-like cell line (KGN, RCB1154), a steroidogenic human granulosa cell line, were purchased from RIKEN Bioresource Centre (Tsukuba, Japan). The physiological characteristics of granulosa cells are maintained in KGN cells [37]. These characteristics include the expression of functional FSH receptors and steroidogenic activity, such as estradiol production in response to FSH stimulation. The purchased KGN cells were cultured in Dulbecco’s Modified Eagle Medium: Nutrient Mixture F-12 (DMEM/F-12) supplemented with 10% fetal bovine serum (Corning, AZ, USA) and 1% antibiotic–antifungal 100 × antibiotic antimycotic solution (Life Technologies, USA) in 5% CO_2_ at 37°C. The cells were seeded at density of 15,000 cells/well in 96-well plates; after incubation for 24 h, the wells were treated with various concentrations (0–12 μg/ml) of 4-hydroperoxycyclophosphamide (4-OOH-CP), which is the active metabolite of CTX (39800-16-3, Cayman Chemical Company, USA).

### Human umbilical vein endothelial cells

Human umbilical vein endothelial cells (HUVECs) were maintained in 10-cm fibronectin-coated dishes containing endothelial cell medium (ScienCell Research Laboratories, USA) supplemented with 5% fetal bovine serum, 50 μg/ml endothelial cell growth supplement, and 1% penicillin–streptomycin solution at 37°C and 5% CO_2_. All experiments were performed using HUVECs at passages 3–5.

### Tube formation assay (thin layer method)

Geltrex LDEV-Free reduced growth factor Basement membrane matrix (Invitrogen, CA, USA) was thawed at 2–8°C overnight. Pre-chilled 24-well plates were coated with the matrix (50 µL/cm^2^ onto the growth surface of each well and incubated for 30 min at 37°C. The cells (2 × 10^4^/cm^2^) were placed in 500 µL DMEM/F12 with various supplements: 10% FBS and endothelial cell growth supplement (positive control group), 20% CM in basal DMEM/F12, and 20% E2-CM in basal DMEM/F12. Anti-angiogenin antibody at 1/100 dilution (sc-74528, Santa Cruz, USA) was preincubated with CM (anti-ANG/CM group) or E2-CM (anti-ANG/E2-CM group) 1 h before tube formation assays were performed. Nonsupplement medium was used for the negative control group. After an additional 8 h of incubation, the endothelial cell tubular structure was formed. Tubes were labeled using 4-µg/ml (approximately 4 µM) Calcein AM Fluorescent Dye (354,217, Corning, USA) in 0.5-ml basal medium for another hour at 37°C. The tube areas from five random fields per well were photographed using a fluorescence microscope (Olympus BX51, Japan). The total tube length and total branching points were measured using the online software WimTube (Onimagin, Spain). This experiment was repeated three times in triplicate. Data are expressed as means ± standard deviations (SDs).

### CTX-induced KGN granulosa cell injury model

To establish an in vitro granulosa cell injury model, the KGN cells were seeded and cultivated upon reaching 50–60% confluence, after which they were treated with 4-OOH-CP (8 µg/ml) for 12 h to induce cell apoptosis and senescence. The 4-OOH-CP-treated KGN cells were cultured with basic medium (DMEM/F12 mixed with 20% MCDB201 medium) and divided into three groups, namely the Mock group (supplemented with basic medium), CM group (supplemented with CM), and E2-CM group (supplemented with E2-CM). After 48 h of incubation, the cells were collected to enable analysis the markers of apoptosis and proliferation. Untreated KGN cells maintained in regular medium served as the control group.

### CTX-induced C57BL/6J POI mouse model

The CTX-induced mouse POI model was established per the protocol described previously [38]. In brief, seven-week-old adult female C57BL/6J mice were purchased from the National Laboratory Animal Center, Taipei, Taiwan. The mice were fed a standard laboratory diet, provided with ad libitum access to water, and subjected to a 12:12-h light:dark cycle. To evaluate the effects of the pcMSC secretome on chemotherapy-induced POI, mice were randomly divided into 4 groups, namely the control, POI, CM, and E2-CM groups. The mice in the POI, CM, and E2-CM groups were intraperitoneally injected with CTX (Sigma-Aldrich, USA) resuspended in normal saline (50 mg/kg/day) for 14 consecutive days to establish a POI model [38], and the mice in the control group were injected with saline. To study the mRNA expression of clock genes in the ovaries and SCN, ovarian and SCN tissue sampling was performed every 4 h for 24 h, starting from Zeitgeber time 0 (ZT0). In each group, we conducted an average of three independent biological replicates per Zeitgeber time (ZT) point (six time points in total). The data for the 0-h and 24-h time points were identical [39]. All animal experiments conducted in the present study were approved by the Institutional Animal Care and Use Committee of Taipei Medical University (approval numbers: LAC-2021-0049 and LAC-2022-0171) and complied per the Guide for the Care and Use of Laboratory Animals.

### Circadian locomotor activity measurement and analysis

We performed actimetry according to established procedures [40, 41]. In brief, the mice were individually housed in specially designed light-sealed boxes equipped with computer-controlled light-emitting-diode lighting and a ventilating fan, as described in the previous subsection [40]. The mice had ad libitum access to food and water, and the light inside the boxes was computer-controlled to match the conditions in the breeding room. Outside the enclosures, the animal room was illuminated solely with safety lights, with computer monitors shielded by safety films. Inside the box, circadian locomotor activity of each mouse was continuously monitored using a passive infrared (PIR) motion sensor and recorded at 1-min intervals. Scheduled light control and data acquisition were managed through an Arduino Mega 2560 microcontroller and custom software [42]. The mice underwent a habituation phase for one week (Week 1) in the boxes under a 12:12-h light:dark (LD) cycle; the lights were turned on at 7:00 a.m. and turned off at 7:00 p.m. Subsequently, daily intraperitoneal (IP) injections of CTX (50 mg/kg) were administered to induce the POI model during Week 2 under LD and Week 3 (constant darkness, DD) of the experiment. Subsequently, in Weeks 4 and 5, the mice in the CM and E2-CM groups received CM and E2-CM treatments every two days under DD. Intensive observation under DD was conducted in Week 6, and observation under an LD cycle was conducted in Week 7. On the final day, the entrainment reverted to DD, and the mice were sacrificed at circadian timepoints for the collection of ovaries and SCN tissues, as well as blood samples. IP injections (Weeks 3–5) and sample collections were conducted under dim safety lighting. Circadian locomotor activities were visualized side-by-side as double plots and spectrograms. Time-dependent period and rhythmicity were assessed using a sliding window fast Fourier transform to generate a circadian heatmap [41]. The resulting data were presented as spectrograms, showing the power spectrum over a range of periods for each time point. During the observation phase of Week 6 (DD), the circadian free-running period was estimated from the peak power in the FFT. The circadian power ratio (CPR) was calculated by dividing the area spanning 20 h and 28 h by the total area of the spectral power in the frequency domain.

### Histology

The collected ovaries were fixed in 10% neutral buffered formalin at 4°C for 24 h. The specimens were then dehydrated, cleared in xylene, embedded in paraffin wax, serially sectioned into 5-μm-thick slices, and mounted on glass slides. Hematoxylin–eosin staining was performed on every tenth sample from three consecutive slides, sampled from a total of 20 ovaries across 4 experimental groups (*n* = 5), followed by blinded follicle counting. Whole-slide scanning at 200 × magnification was carried out using TissueFaxs (TissueGnostics GmbH, 1020 Vienna, Austria) for analysis. Follicles with a visible nucleus were counted and classified as primordial, primary, secondary, antral, or atretic. A primordial follicle was identified as an oocyte surrounded by a single layer of flat pregranulosa cells. Primary follicles had a single layer of cuboidal granulosa cells, whereas secondary follicles had multiple layers of cuboidal granulosa cells. In antral follicles, an antrum was present in the granulosa cell layers. Atretic follicles were identified by eosinophilia of the ooplasm, nuclear pyknosis of granulosa cells, cytoplasmic contraction, cytoplasmic vacuoles, and the dissociation of granulosa cells from the basal membrane [43]. To evaluate ovarian fibrosis, modified Masson’s staining was performed using the Modified Masson’s Trichrome Stain Kit (ScyTek Laboratories, USA). Specifically, tissue slides were cut from paraffin-embedded blocks, randomly selected (*n* = 4), then subjected to deparaffinization, rehydration, and staining according to the manufacturer's protocol. After mounting with resin, slides were photographed at 200 × magnification. ImageJ software was used to quantify the area of fibrosis indicated by collagen deposition (stained blue).

### RNA isolation and real-time quantitative polymerase chain reaction

Ovarian and SCN tissues, pcMSCs, and KGN cells were collected and homogenized, and their total RNA was isolated using an EasyPure Total RNA Spin Kit (Bioman Scientific, Taiwan) per the manufacturer’s instructions. RNA quantity and quality were assessed using a NanoDrop spectrophotometer (Thermo Scientific, USA).

We synthesized cDNA using Moloney murine leukemia virus reverse transcriptase (M-MLV RT, M1705, Promega, USA). Ovary RNA (2000 ng) and SCN RNA (400 ng) were mixed with a reverse transcriptase master mix in a final volume of 25 μL. The reaction mixtures were incubated at 25°C for 5 min, 50°C for 60 min, and 70°C for 10 min.

Real-time quantitative polymerase chain reaction (PCR) was performed in 96-well plates on a LightCycler 96 (Roche Diagnostics, Switzerland) by using the Fast SYBR Green Master Mix (15,350,929, Applied Biosystems, MA, USA). PCR reactions were conducted using a mixture comprising 5 μL of SYBR Green I Master, 2.5 μL of RNAse-free water, 2 μL of 30 nM primer mix, and 0.5 μL of cDNA for a total volume of 10 μL. Three technical replicates were performed for each sample. The cycling conditions were as follows: an initial cycle was performed for 20 s at 95°C, followed by 40 cycles for 10 s at 95°C, 20 s at 60°C, and 20 s at 72°C. Melting curve analyses for determining the dissociation of PCR products were performed at between 65 and 95°C. The PCR efficiencies of the primers was evaluated by examining the samples through the use of six standards in triplicate (diluted in fivefold series). Two reference genes (*Actb* and *Gapdh*) were used for normalization when the expression of the clock genes was analyzed. Cq values were exported using the software LightCycler 96 (Roche Diagnostics, Switzerland) and analyzed using Microsoft Excel. The PCR efficiency factor (1 denoting 100% efficiency) and Cq values were quantified without any weighting. A PCR efficiency of 90–110% was regarded as acceptable [39]. Table S1 lists the primer sequences for the targeted genes in the real-time quantitative PCR.

### Western blot analysis

The collected cells and ovaries were lysed with RIPA buffer (Energenesis Biomedical, Taiwan) along with a protease inhibitor cocktail (Roche Diagnostics, Switzerland) and phosphatase inhibitor cocktail (Roche Diagnostics). Total protein solutions were obtained by centrifuging the lysed samples at 14,000 rpm for 25 min at 4°C. The protein concentration was detected using the Pierce BCA assay kit (Thermo Fisher Scientific, USA) per the manufacturer’s instructions. Equal amounts of protein (30 µg) in sodium dodecyl sulfate (SDS) buffer were separated by applying electrophoresis to 10% SDS–polyacrylamide gel, after which the separated proteins were transferred to pure polyvinylidene fluoride membranes (Immobilon, Merck Millipore, Germany). The membranes were placed in 5% skimmed milk for 1 h to block nonspecific binding and were then incubated with corresponding primary antibodies (Table S2). They were then washed three times with tris-buffered saline (TBS) buffer (Bioman, Taiwan) containing 0.2% Tween 20 (Bioshop, Canada). After undergoing rinsing, the membranes were incubated with anti-rabbit or anti-mouse IgG secondary antibody (diluted 1:3000). Finally, they were visualized using Immobilon Western (Millipore, Germany) and imaged using the ImageQuant LAS 4000 mini system (GE Healthcare, USA). Band density was quantified using ImageJ software 1.53u (NIH Image, USA), β-actin was used as an internal control.

### Immunohistochemical and immunocytochemical staining

Slides were incubated in a heating chamber at 60°C for 30 min, immersed in xylene, and rehydrated through a graded alcohol series. Deparaffinized sections were treated with 10 mM citrate buffer (pH 6.0) for 20 min at 98°C for antigen retrieval and were subsequently washed with TBS and permeabilization with TBST (0.2% Tween 20 in TBS solution). These sections were then blocked with 5% (v/v) normal goat serum (Vector Laboratories, S-1000, USA) for 1 h. After blocking, the slides were incubated overnight with the following primary antibodies: CYP19A1 (Abcam, ab18995, UK), PER2 (Abcam, ab227727, UK), RORA (Proteintech, 10616-1-AP, USA), and Rev-Erbα (NR1D1, Proteintech, 13906-1-AP, USA). Subsequently, they were incubated with goat anti-rabbit horseradish peroxidase–conjugated secondary antibody (Vector Laboratories, USA) for 1 h. Finally, the sections were stained with 3,3’-diaminobenzidine substrates (Vector Laboratories, USA).

For confocal fluorescence detection, the slides were subjected to incubation overnight with mouse anti-PCNA (Chemicon, CBL407, Germany) or double staining with cleaved caspase-3 (Cell Signaling, #9961, USA) by using the In Situ Cell Death Detection Kit, POD (TUNEL assay, Roche, Cat. No.11684817910, Switzerland). Double immunofluorescence staining was also performed using antibodies against CD31 (550274, BD Pharmingen, USA) and VEGF-A (ab183100, Abcam, UK). On the next day, after being washed with TBST, the sections were incubated for 1 h at room temperature with Alexa Fluor-594–labeled goat anti-mouse IgG/goat anti-rabbit IgG (Life Technologies, USA) and then counterstained with DAPI (4',6-diamidino-2-phenylindole). Fluorescence signals were detected using a Stellaris 8 confocal microscope (Leica, Germany).

KGN cell cultures grown on glass coverslips were fixed with 4% newly prepared paraformaldehyde for 10 min at room temperature, permeabilized with cold TBST for 10 min, and incubated with a blocking solution containing 5% normal goat serum in TBST for 1 h. The coverslips were incubated overnight at 4°C with primary antibodies for the proteins: Ki67 (Abcam, ab15580, UK) and cleaved caspase-3 (Cell Signaling, #9961, USA). Subsequently, the coverslips were incubated with goat anti-rabbit Alexa Fluor 488-conjugated IgG (Life Technologies, USA), and F-actin was labeled with Alexa Fluor 594 phalloidin (Invitrogen, A12381, USA). Fluorescence signals were detected using a confocal Stellaris 8 microscope (Leica, Germany). Table S2 presents the experimental conditions for the antibodies used for Western blotting or immunostaining.

### Enzyme-linked immunosorbent assay

To perform enzyme-linked immunosorbent assays (ELISA), serum samples were obtained from the mice in each experimental group to evaluate the levels of AMH (MBS2507173, MyBioSource, CA, USA), E2 (MBS261250, MyBioSource, CA, USA), and FSH (MBS2507988, MyBioSource, CA, USA). Fresh CM and E2-CM from four consecutive passages were used to quantify angiogenin (ELH-ANG, Raybiotech, GA, USA). All assays were conducted using ELISA kits according to the manufacturers’ instructions.

### Flow cytometric analysis

KGN cells were treated with Allophycocyanin (APC) Annexin V in a staining buffer containing propidium iodide (BioLegend, USA) per the manufacturer’s recommendations. The samples were analyzed using a BD FACSVerse system and the software BD FACSuite (CA, USA).

### Cytokine array assay

The cytokines in CM/E2-CM were detected using antibody array-based technology (Human Cytokine Arrays C5, RayBiotech, USA). Each array was incubated with CM/E2-CM (700 µg/ml) at 4°C overnight in accordance with the manufacturer’s instructions. The signals on the membranes were imaged using the ImageQuant LAS 4000 mini system (GE Healthcare, USA). Integrated densities were measured using ImageJ 1.53u. The samples were normalized on the basis of six spots of positive controls and the background of the surrounding area.

### Exosome isolation and characterization

To obtain the exosomes in CM and E2-CM, the ER^+^pcMSCs were cultured under serum-free conditions with or without E2 priming until they reached 80% confluence, after which they were maintained in new serum-free medium for 48 h. Cell debris was removed by centrifuging the samples at 300×g for 10 min at 4°C. The supernatant was filtered through membranes (pore size: 0.22 μm). The exosomes were isolated through the following steps. The medium was ultracentrifuged at 100,000×g at 4°C for 90 min (Optima L-90 K Ultracentrifuge, Beckman Coulter, USA), the supernatant was carefully collected into another tube, and the pellet was resuspended in 10 ml iced PBS. The suspension was ultracentrifuged again at 100,000×g and 4°C for 90 min, and the supernatant was carefully removed. The final pellets were resuspended in 250 μL iced PBS as crude exosomes. The exosomes present in fresh conditioned media (CM and E2-CM) were quantified by employing digital exosome counting technology (JVC Exocounter, Japan), in which anti-CD9 and anti-CD63 antibodies are used for exosome labeling. The system excludes larger particles, such as microvesicles, through a size-selective nano-structure on a disc with a 260-nm width. Each exosome is labeled with a single nanobead and detected through optical pickup, which was developed on the basis of Blu-ray technology.

### Exosomal miRNA extraction, library preparation, sequencing and qPCR

Total RNA was extracted using TRIzol LS reagent (10296010, Invitrogen, CA, USA) per the manufacturer’s instructions. The RNA concentration was determined by using a spectrophotometer (ND-1000, NanoDrop Technology, USA) to measure absorbance at 260 nm. The quantity of RNA was evaluated by using a LabChip RNA 6000 kit (Agilent Technologies, USA) and the Bioanalyzer 2100 (Agilent Technology). Sample libraries were prepared using the QIAseq miRNA Library Kit (Qiagen, Germany) per the manufacturer’s guidelines, and subsequently they were sequenced using an Illumina instrument (75-cycle single-end read, 75SE). The miRNA data obtained in the present study have been deposited in the Gene Expression Omnibus database under the accession number GSE247568.

The collected sequencing data were analyzed using the Illumina BCL2FASTQ v2.20 software (Illumina, USA) for demultiplexing. We used Trimmomatic to isolate high-quality reads, discarding those shorter than 18 nucleotides [44]. The processed data were then analyzed using miRDeep2 [45]; during this process, the reads were aligned with the GRCh38 reference genome obtained from the University of California, Santa Cruz [46]. Given that human miRNAs can only align with a limited number of genomic locations, we only considered reads that matched the genome up to five times for miRNA identification [47]. We employed reads per million mapped reads to measure normalized miRNA expression. This model was derived by dividing the signal values of each miRNA by the total number of mapped reads. We used the MirTarget V4 tool [48] of miRDB version 6 [49] to predict miRNA targets. For this process, we only included functional human miRNAs from the FuncMir collection (http://www.mirdb.org/FuncMir.html). In addition, when searching multiple miRNAs or genes for target mining, we excluded gene targets with fewer than 60 target prediction scores and miRNAs with more than 2000 predicted targets in the genome. TargetScan (v8.0; targetscan.org [50, 51]) was employed to screen for miRNAs targeting genes of interest, with selection based on a TargetScan context +  + score ≤ − 0.2. For the differentially expressed miRNA analysis, we estimated the read counts of mature miRNA obtained from miRDeep2 Quantifier module. Then the read counts of each miRNA were normalized to total number of miRNAs as RPM (Reads per millions mapped reads) to compare the abundance between samples.

Total exosomal RNA extracted above, including miRNA, was used to conduct quantitative analysis of miRNA by qPCR. cDNA was synthesized using the miRCURY LNA RT Kit (Cat#339340, Qiagen, Hilden, Germany) according to the manufacturer’s recommendation. Reverse transcription reactions were performed using the miScript HiSpec Buffer in the kit, allowing selective conversion of mature miRNAs but not precursors. Mature miRNAs were polyadenylated by poly(A) polymerase and reverse transcribed into cDNA using oligo-dT primers. Polyadenylation and reverse transcription were performed in parallel in the same tube. The expression of the miRNAs of interest was assessed via qPCR using the miRCURY LNA SYBR Green PCR kit (Cat#339345 Qiagen, Hilden, Germany). U6 (RNU6-1) snRNA was chosen as the internal control for microRNAs. Primers were designed based on a miRNA-specific stem loop-RT assay [52]. Each experiment was performed at least in triplicate. Primer sequences for miRNAs are shown in the supplementary table (Table S3). Relative gene expression was calculated using the 2^−ΔΔCq^ method.

### Statistical analysis

All experiments were performed at least three times. The collected data were analyzed using GraphPad Prism (version 9.3, USA) and were presented as means ± SDs. An unpaired Student’s *t* test was conducted to perform between-group comparisons. One-way analysis of variance (ANOVA) and two-way ANOVA with Tukey’s test were performed to compare the results of multiple groups. A *p-*value < 0.05 was considered to indicate significance.

## Results

### Establishment of a CTX-induced POI mouse model

The effects of CTX on the POI-associated ovarian morphology, sex hormones, and mouse body weight were analyzed. As presented in Supplementary Fig. 1, the CTX-induced POI mice exhibited significant weight loss (Fig. S1A), reduced ovary size, poor ovarian morphology (Fig. S1B), impaired folliculogenesis (Fig. S1C and D), decreased serum E2 and AMH levels, increased serum FSH levels (Fig. S1E), and decreased CYP19A1 protein levels (Fig. S1F). These results indicate the successful establishment of a CTX-induced POI mouse model.

### Expression of ERα of pcMSCs

Estrogen is produced by granulosa cells. It stimulates ovarian folliculogenesis and plays a key role in the interaction between granulosa cells and surrounding niche cells [53, 54]. The MSCs expressing ERs may respond better to estrogen in the ovarian niche for effective POI treatment. The results of a Western-blot analysis revealed that the pcMSCs expressed higher levels of ERα relative to those of the MSCs from other tissues from the umbilical cord, adipose tissues, and bone marrow. MCF-7 (ER^+^ breast cancer cells) and MDA-MB-231 (triple negative breast cancer cells) served as positive or negative control (Fig. S2A). E2 treatment increased the cell proliferation (WST1 assay, Fig. S2B-a) and the protein levels of proliferating cell nuclear antigen (PCNA; Fig. S2B-b), highlighting the active estrogen-ER signaling of the pcMSCs in the ovarian niche.

### Conditioned medium derived from ER^+^pcMSCs with or without E2 priming (E2-CM and CM, respectively) improved ovarian function in CTX-induced POI mouse model

We previously demonstrated that pcMSC (same source as ER^+^pcMSCs in this study) exosomes can mitigate endoplasmic reticulum stress, inflammation, and lung injury in lipopolysaccharide-treated obese mice [55] and mitigate liver injury in high-fat diet–induced obese mice [56]. In the present study, we enhanced the response to ovarian estrogen niche and investigated the effect of the ER^+^pcMSC secretome on POI treatment; CM derived from ER^+^pcMSCs without or with E2 priming (100 nM for 48 h) was collected for experiments (CM and E2-CM, respectively).

**Fig. 1:**
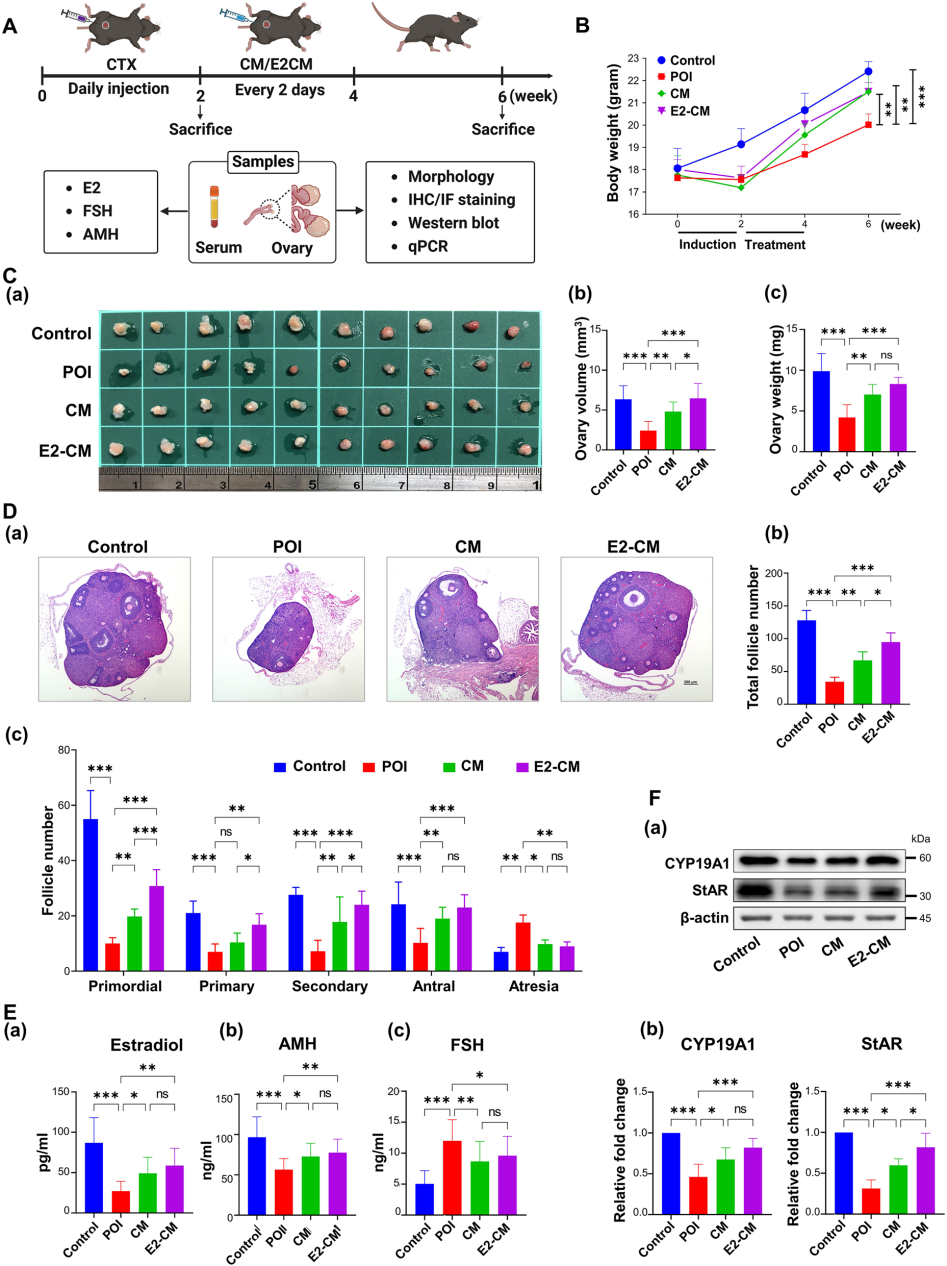
pcMSC-CM and E2-CM improved ovary function in CTX-induced POI animal model. **A** Experimental schedule and design. **B** Changes in bodyweights of mice in four experimental groups (control, POI, CM, and E2-CM groups).** C** Tissue morphology (**a**), volume (**b**), and weight (**c**) of ovaries of mice in four experimental groups (*n* = 10); volume (mm^3^) = length × width^2^ × π/6. **D** (**a**) HE staining results indicate folliculogenesis in ovary tissues of mice in each group. Quantitative analysis of (**b**) total follicle number and (**c**) follicle number at various folliculogenesis stages in each group (*n* = 5). **E** Serum hormone levels of E2 (**a**), FSH (**b**), and (**c**) AMH in each group. Results obtained through ELISA assay. **F** Protein levels of steroidogenesis pathway (CYP19A1 and StAR), as detected using Western-blot assay. Data are expressed as means ± SDs of at least three independent experiments. *p < 0.05, **p < 0.01, ***p < 0.001 compared with control group. One-way and two-way ANOVA with Tukey’s test

The mice were divided into four groups, namely the control group comprising healthy controls, the POI group comprising CTX-induced POI mice, the CM group comprising POI mice treated with CM, and the E2-CM group comprising POI mice treated with E2-CM. The POI mice were intraperitoneally injected with PBS, CM, or E2-CM. The experimental designs are presented shown in Fig. 1A. Relative to the mice in the control group, those in the POI group exhibited significant decreases in body weight (Fig. 1B) and ovary size (Fig. 1C). In both the CM and E2-CM groups, the treatments had a significant therapeutic effect on both the body weight (Fig. 1B) and ovary size as well as the ovary weight (Fig. 1C) of the CTX-induced POI mice. By examining every tenth slide from three consecutive slides of each ovary, sampled from a total of 20 ovaries across 4 experimental groups, we found that treatment with CM or E2-CM improved folliculogenesis in the CTX-induced POI mice, as evidenced by increases in the total number of follicles (Fig. 1D, Panels a and b) and in the number of follicles from the primordial to antral developmental stages. After treatment with CM or E2-CM, the atretic follicles decreased whereas the number of primordial follicles increased (Fig. 1D, Panel c). Furthermore, the mice in the CM and E2-CM groups exhibited increased E2, decreased FSH levels, and restored ovarian reserves (as evidenced by their elevated AMH levels; Fig. 1E). Besides, CM and E2-CM treatment increased the levels of steroidogenesis-related proteins (CYP19A1 and StAR; Fig. 1F). The treatment effects were more prominent in the mice in the E2-CM group than in those in the CM group (Fig. 1C, D, and F). Collectively, these results reveal the therapeutic effects of ER^+^pcMSC-CM and E2-CM on restoring folliculogenesis and steroidogenesis in the ovaries of the CTX-induced POI mice.

### ER^+^pcMSC-derived CM and E2-CM reduced granulosa cell apoptosis in the CTX -induced POI mouse model

**Fig. 2:**
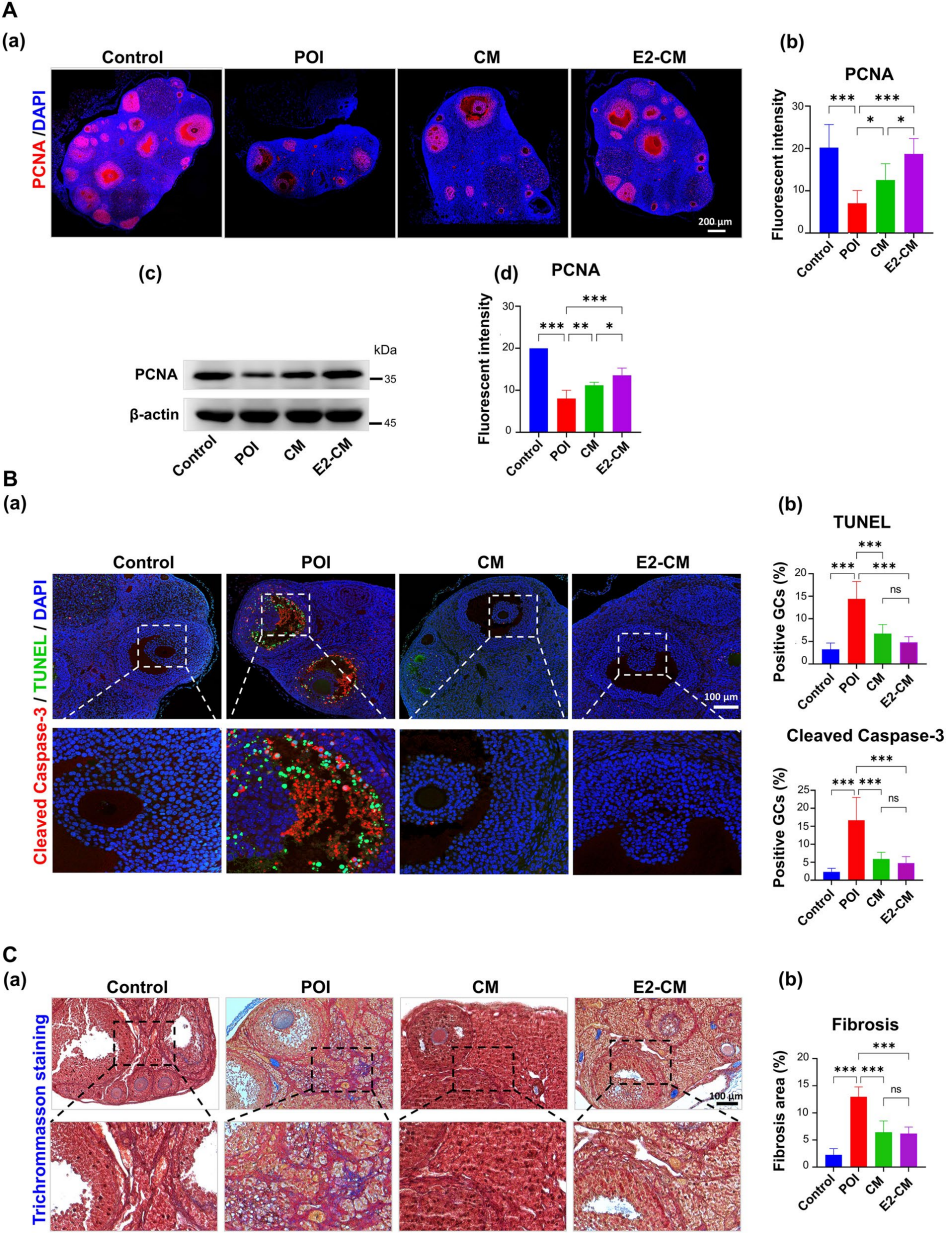
pcMSC-CM and E2-CM protected GCs from apoptosis in CTX-induced POI animal model.** A** Effect of CM and E2-CM treatment on ovarian cell proliferation was analyzed using a confocal microscope (**a, b**) and Western blotting (**c, d**). **B** Effects of CM and E2-CM on CTX-induced cell death and apoptosis of GCs. Results obtained through TUNEL assay. TUNEL (apoptotic cells) results presented in green; cleaved caspase-3 results in red; and DAPI results in blue. **C** Trichrome (modified Masson’s staining) results reveal fibrotic structure (muscle fiber, in dark red) and collagen accumulation (in blue) (*n* = 4). Data are expressed as means of at least three replicates ± SDs. *p < 0.05, **p < 0.01, ***p < 0.001. One-way ANOVA with Tukey’s test

To determine the effects of CM/E2-CM on ovarian damage due to CTX-induced POI, immunohistochemical staining for PCNA was performed; PCNA is an indicator of cell proliferation. As revealed in Fig. 2, compared with the healthy controls, which exhibited high PCNA expression in granulosa cells, the POI group exhibited a remarkable loss of PCNA expression. Treatment with ER^+^pcMSC-CM or E2-CM effectively restored the expression of PCNA in the granulosa cells, as evidenced by the consistent positive results for immunohistochemical staining that were obtained through immunoblotting (Fig. 2A, Panels a and b vs. Panels c and d). Furthermore, terminal deoxynucleotidyl transferase dUTP nick end (TUNEL) assay and cleaved caspase-3 double immunostaining were performed to determine the effect of CM or E2-CM on decreasing granulosa cell apoptosis in the CTX-induced mice. Figure 2B reveals that CTX induction increased both the positive TUNEL (cell death) and expression of cleaved caspase-3 protein (apoptosis) in the POI group. That is, CM and E2-CM helped to prevent the cell death/apoptosis of granulosa cells in the POI groups. The Masson’s trichrome staining results revealing the effects of CM and E2-CM on CTX-induced ovarian tissue fibrosis are presented in Fig. 2C. These results clearly indicate that ER^+^pcMSC-CM and E2-CM enhanced the proliferation and reduced the apoptosis of granulosa cells in the CTX-induced POI mice.

### ER^+^pcMSC-derived CM and E2-CM reduced granulosa cell apoptosis in the CTX-challenged KGN cell model

**Fig. 3:**
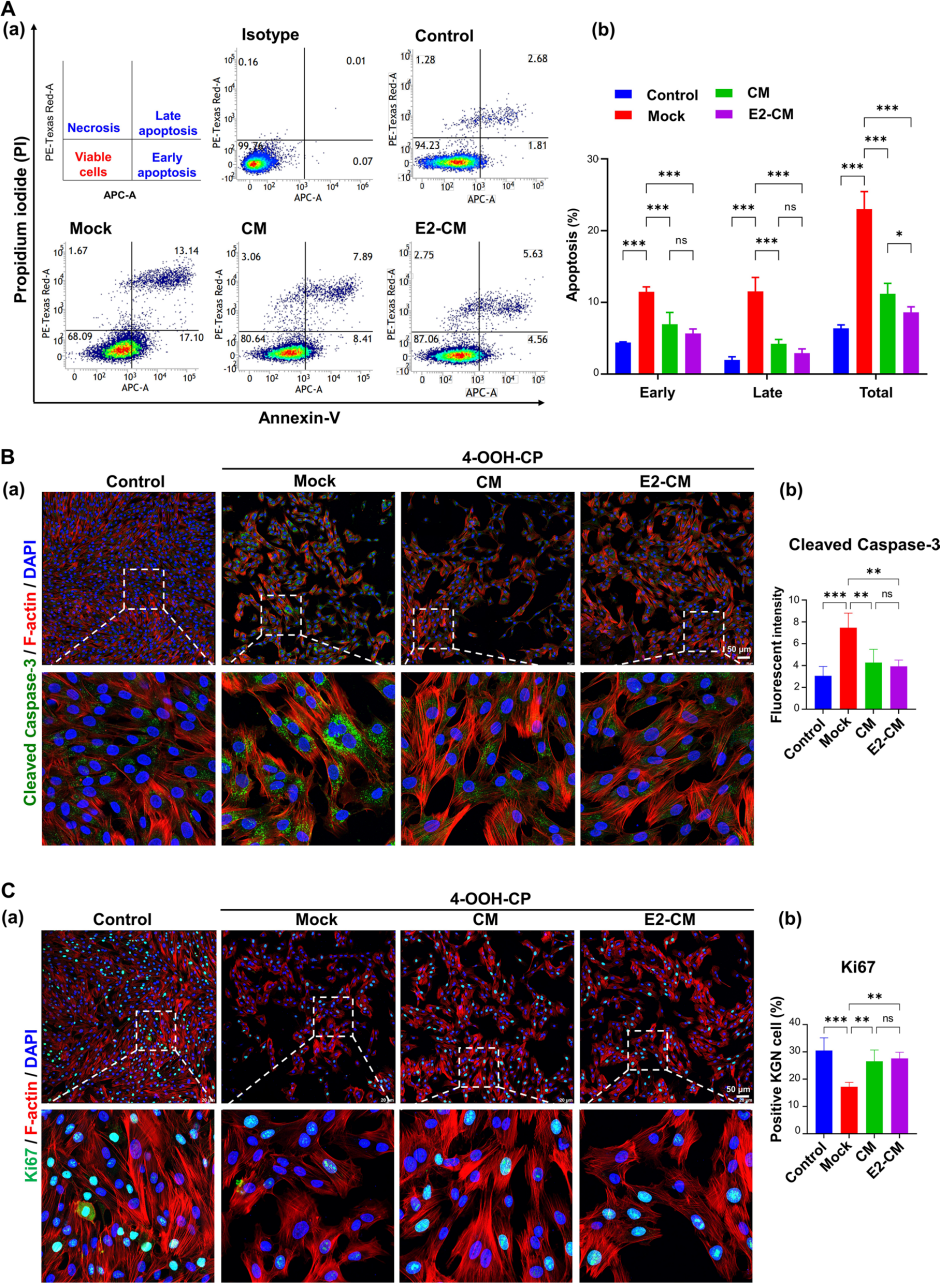
pcMSC-CM and E2-CM restore granulosa cell viability in CTX-challenged KGN cell model. **A** Apoptosis of KGN cells in four experimental groups: control, Mock (4-OOH-CP treatment), CM (4-OOH-CP treatment plus CM), and E2-CM (4-OOH-CP treatment plus E2-CM) (**a**). Results obtained through flow cytometry analysis. Results of quantitative analysis of (**a**) are presented in (**b**) (*n* = 3).** B** Levels of cleaved caspase-3 (in green), actin filaments (in red), and DAPI (in blue) (*n* = 4). **C** Ki67 levels in KGN cells (*n* = 4). Results obtained through confocal immunofluorescent analysis. Results of quantitative analysis of (**a**) are presented in **(b).** *p < 0.05, **p < 0.01, ***p < 0.001. One-way ANOVA with Tukey’s test

To elucidate the effects of CM and E2-CM on granulosa cell apoptosis, the human KGN granulosa cell line was used to establish an in vitro cell model (Fig. S3A). In the present study, 4-OOH-CP (8 μg/ml), an active form of CTX, was employed to test the effects of CM and E2-CM (Fig. S3B). The flow cytometry analysis results reveal that, when compared with the 4-OOH-CP-treated KGN cells (Mock group), those treated with CM or E2-CM exhibited significantly reduced 4-OOH-CP-induced early and late cell apoptosis (Fig. 3A, *n* = 3 for each group). This finding was further confirmed by immunostaining for cleaved caspase-3 protein (Fig. 3B). Additionally, CM or E2-CM treatment also significantly rescued the 4-OOH-CP-suppressed Ki67 expression, cell proliferation, and viability of the KGN cells (Fig. 3C, Fig. S3C and S3D). Collectively, these results demonstrated the antiapoptotic effects of ER^+^pcMSC-CM/E2-CM on 4-OOH-CP-induced KGN cells, with E2-CM exhibiting superior efficiency relative to CM (Fig. 3).

### ER^+^pcMSC-derived CM and E2-CM restored ovarian circadian clock and locomotor activity in CTX-induced POI mouse model

Estrogen is closely associated with circadian rhythm regulation [19], and the ovarian circadian clock has been reported to regulate ovarian function [20, 26, 57–59]. Given that the CTX-induced POI mice exhibited decreased E2 levels and that CM and E2-CM treatment effectively and significantly restored CTX-induced E2 defects (Fig. 1), we hypothesized that CM and E2-CM can regulate the estrogen-associated ovarian circadian clock. To test our hypothesis, the experimental mice were divided into four groups; namely the Control, POI, CM and the E2-CM as described above. We analyzed the expression of genes related to estrogen synthesis and circadian rhythm regulation [60, 61].

**Fig. 4:**
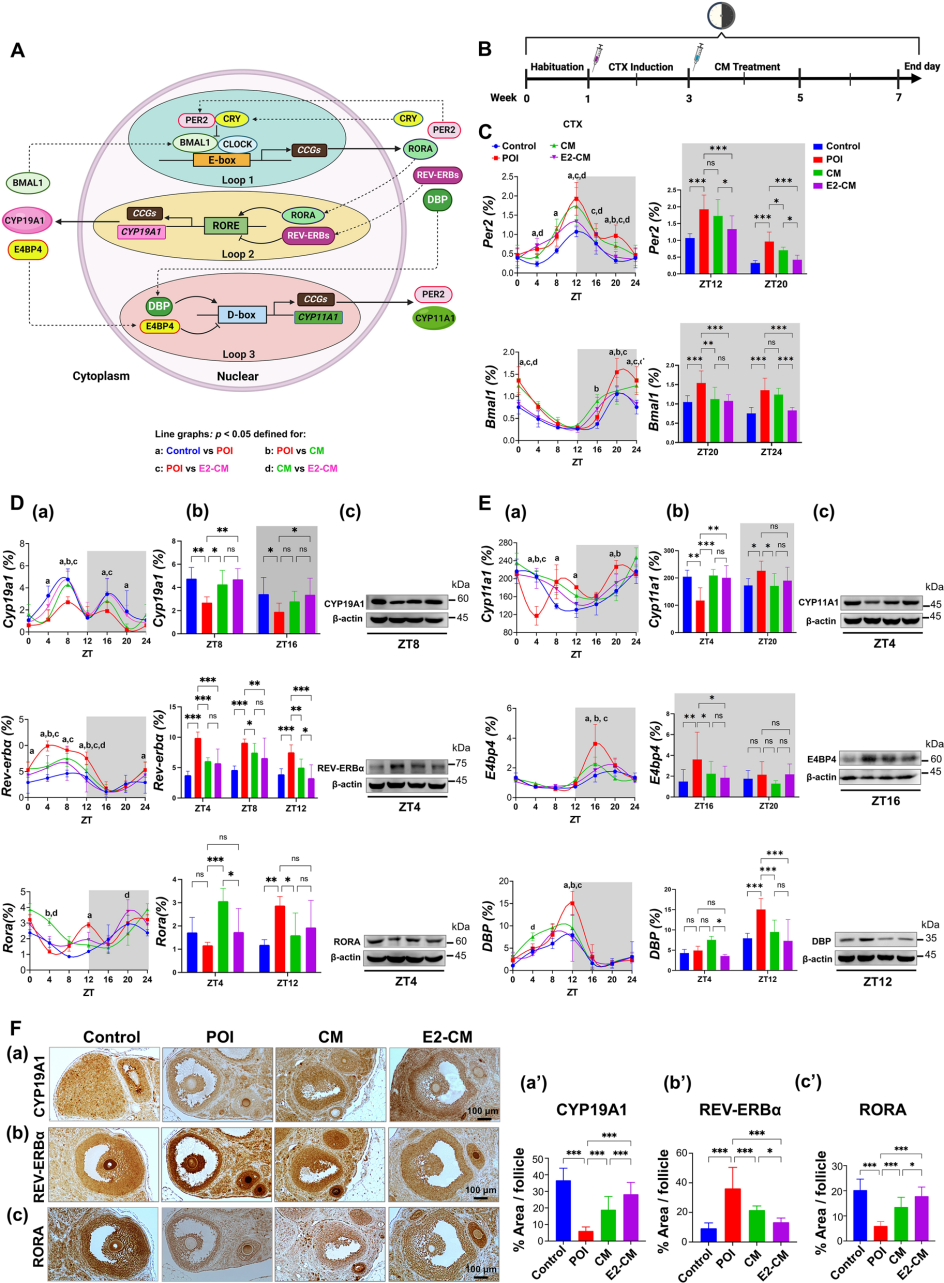

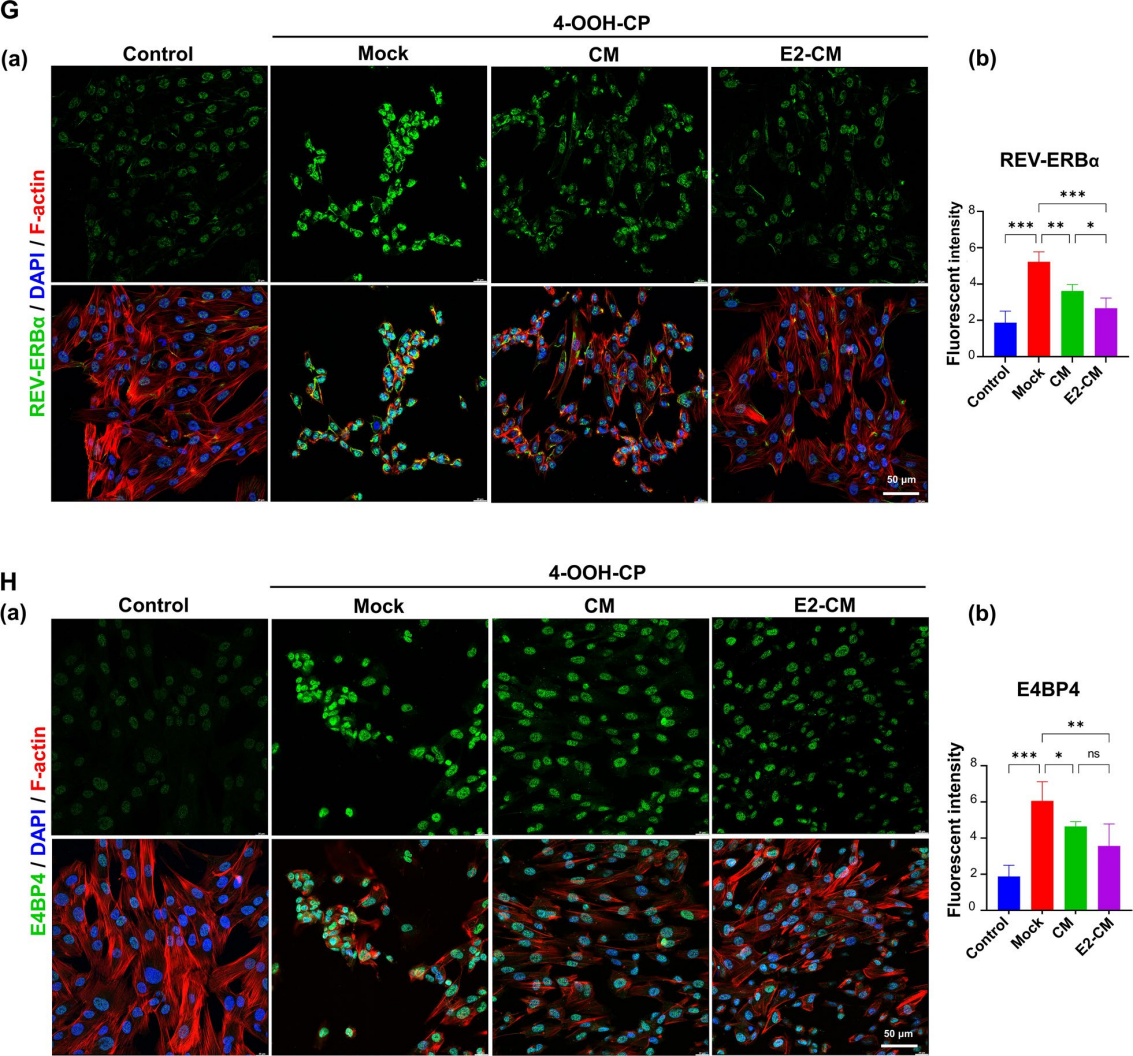
pcMSC-CM and E2-CM restore ovarian circadian rhythm in CTX-induced POI animal model. **A** Regulatory expression loops of *Per2-Bmal1* (Loop1), *Cyp19a1* (Loop 2), and *Cyp11a1* (Loop 3). Clock-controlled elements (CCEs) of *Cyp19a1* and *Cyp11a1* are RRE and D box, respectively. RORA and REV-ERBα competitively bind to RRE (*Cyp19a1*), and DBP and E4BP4 competitively bind to D box (*Cyp11a1*). Binding of RORA and DBP to their targeted CCEs induces upregulation of their targeted genes; this effect is reversed for REV-ERBα and E4BP4. **B** Experimental conditions are illustrated. Four experimental groups were involved, namely the control, POI, CM (POI plus CM), and E2-CM (POI plus E2-CM) groups. **C** Loop 1 for *Per2* and *Bmal1* expression. **D** Loop 2 for expression of *Cyp19a1*-*Rora*-*Rev*-*erbα*. **E** Loop 3 for expression of *Cyp11a1-E4bp4-Dbp*. **a** Gene expression levels detected by RT-qPCR, **b** quantitative and statistical analysis of gene expression (*n* = 3 each time point), and **c** protein levels detected through western blotting at specific ZTs were shown. Curves were created using Fit Spline/LOWESS (medium smoothing). *p < 0.05, **p < 0.01, ***p < 0.001. Comparisons between groups at a given time point (*p* < *0.05*), specifically, **a** between control and POI groups, **b** between CM and POI groups, **c** between E2-CM and POI groups, and **d** between CM and E-CM groups. Two-way ANOVA with Tukey’s test. Light and dark backgrounds represent light and dark phases of the day, respectively. ZT0 indicates the time when the light is switched on, and ZT12 indicates the time when the light is switched off. **F** Protein expression localization and statistical analysis of CYP19A1 (**a** and **a’**), REV-ERBα (**b** and **b**’) and RORA (**c** and **c’**) in ovary tissues of mice in each experimental group (*n* = 5). Results obtained through immunohistochemical staining. **G** and **H** Nuclear expressions of REV-ERBα and E4BP4 in KGN cells were shown. Protein expression levels detected by immunofluorescence staining (*n* = 4). **F**, **G** One-way ANOVA with Tukey’s test

Figure 4A depicts the regulatory loops of gene expressions associated with circadian rhythm and estrogen synthesis, and Fig. 4B illustrates the experimental procedure. Because the SCN is the central pacemaker of the body’s circadian rhythm [19], we analyzed the circadian gene expression of core clock genes such as *Per2* (period circadian regulator 2) and *Bmal1* (basic helix-loop-helix ARNT-like 1). Figure 4C presents potential CTX-induced disruption in the expression of these genes. Compared with the control group, the POI group exhibited a significant increase in *Per2* expression) at the peak ZTs of ZT12 and ZT20, whereas *Bmal1*expression increased during the circadian night at ZT20 and ZT24 (red line vs. blue line). Treatment with CM and E2-CM effectively rescued the CTX-induced expression defects of *Per2* and *Bmal1* in the ovarian tissues of the POI mice (green and purple lines vs. red line).

We analyzed the gene expression related to estrogen biosynthesis (*Cyp19a1* and *Cyp11a*) and circadian rhythm regulation (Fig. 4D). Compared with the control group, the POI group showed a significant decrease in *Cyp19a1* (aromatase) expression (Panel a; ZT8 and ZT16), and CM and E2-CM effectively rescued the *Cyp19a1* expression defect in the treated groups at both the transcript and protein levels (Fig. 4D, Panels b and c). *Cyp19a1* did not exhibit a diurnal rhythm. In line with the previous results, E2-CM treatment was more effective than CM treatment (at ZT16). Additionally, the expression of *Rev-erbα (Nr1d1)* was considerably higher in the POI group than in the control group. Because REV-ERB*α* has been reported to target the ROR/REV-ERB-response element (RRE) by which *Cyp19a1* expression is regulated, the increased *Rev-erbα* may explain the decreased expression of *Cyp19a1* mRNA. By contrast, *Rora* expression exhibited an opposite phase relative to *Rev-erbα*, which can be explained by the role that RORA plays in positively regulating estrogen synthesis in granulosa cells by targeting the RRE on *Cyp19a1*. Notably, compared with the control group and the CM and E2-CM groups, the POI group did not present a diurnal rhythm in *Rora* expressions (Fig. 4D, Panel a). Protein levels of CYP19A1, REV-ERB*α*, and RORA in each experimental groups were further validated through immunoblotting (Fig. 4D, Panel c).

Moreover, cells expressing *Rev-erbα* and *E4bp4* (Fig. 4D vs. 4E, Panel a) exhibited opposite circadian rhythm phases relative to those expressing *E4bp4* and *Dbp* (Fig. 4E, Panel a). REV-ERB*α* is known to repress *E4bp4* to activate NAMPT-dependent NAD^+^ biosynthesis and sustain cardiac function [62]. By contrast, E4BP4 represses a D-box-like DNA binding sequence (DBS) [63], which regulates *Cyp11a1* expression [64]. These findings corroborate our observations of an opposite circadian rhythm phase in cells that express both *E4bp4* and *Dbp* (Fig. 4E, panel a).

As was true with *Cyp19a1* expression, the POI group showed a significant decrease in *Cyp11a1* expression at ZT4 relative to the control group, resulting in a complete loss of the rhythmic pattern. Consistently, *E4pb4* expression increased in the POI group at ZT16. Accordingly, the fluctuation of *Dbp* became stronger in the POI group at ZT12. CM and E2-CM effectively restored the expression defects of *Cyp11a1*, *E4bp4*, and *Dbp* in the treated groups (Fig. 4E, Panels a and b). The protein levels of CYP11A1, E4BP4, and DBP in each experimental group were further confirmed through immunoblotting (Fig. 4E, Panel c).

The impact of CM and E2-CM on the expression of circadian proteins REV-ERBα and E4BP4 in ovarian granulosa cells was further validated through immunofluorescence staining using KGN cells in vitro. Our findings revealed that the CTX analog 4-OOH-CP significantly increased nuclear REV-ERBα levels, while CM/E2-CM effectively attenuated this effect of 4-OOH-CP (Fig. 4G). Similar trends were observed in the expression of E4BP4 (Fig. 4H). These results provide strong support for the observations made in the in vivo animal model as illustrated in Fig. 4D and F.

**Fig. 5:**
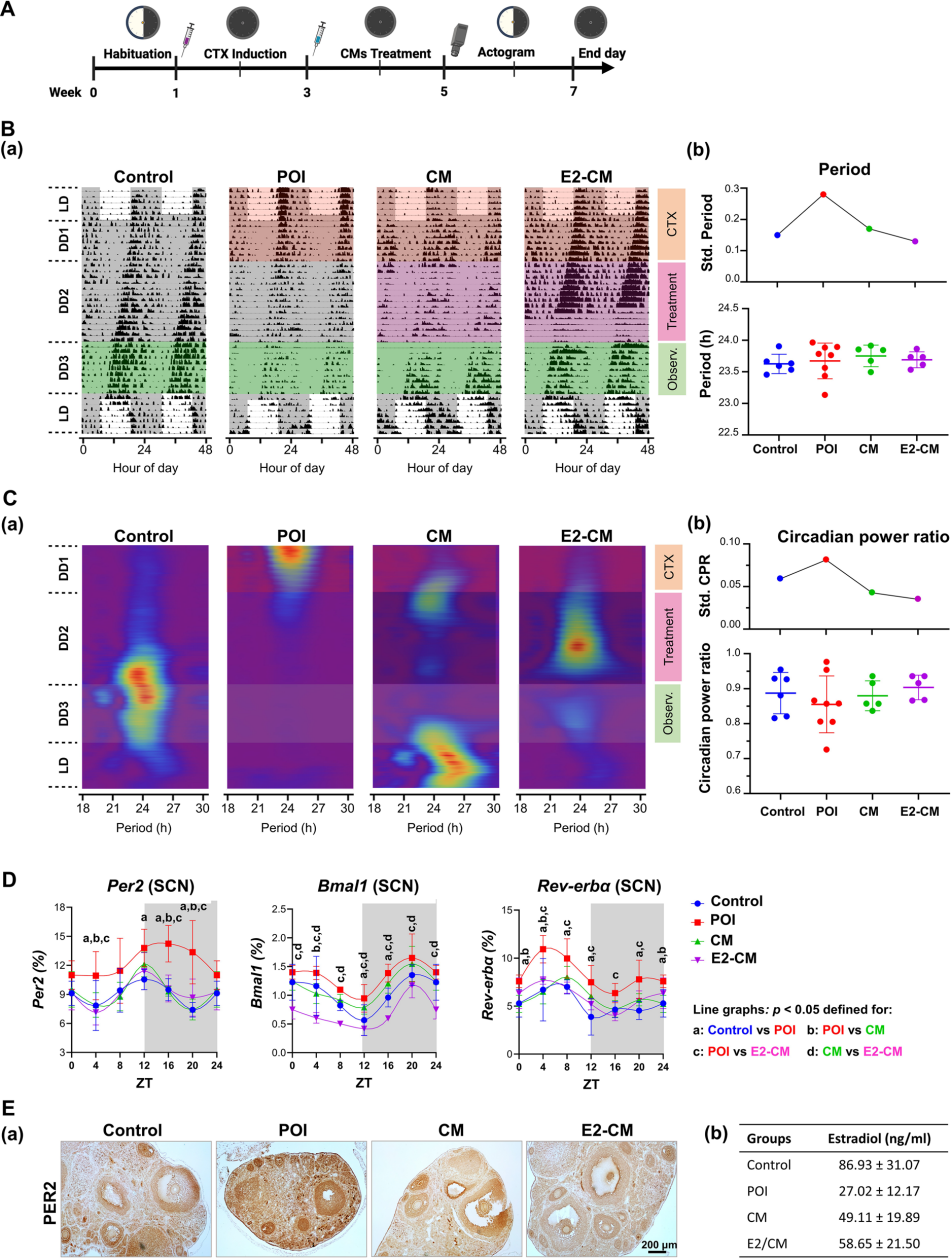
pcMSC-CM and E2-CM restore period in locomotor activity in CTX-induced POI animal model. **A** Experimental conditions are presented. Four experimental groups, namely the control (*n* = 6), POI (*n* = 8), CM (POI plus CM, *n* = 5), and E2-CM (POI plus E2-CM, *n* = 5) groups, were used. LD (L12:D12); DD (D12:D12). **B** Double-plotted actograms from four representative mice, entrained under LD and DD conditions, with gray shading indicating darkness during three experimental phases: CTX, Treatment (Control, CM, or E2-CM), and Observation (Observ.) (**a**). **b** Periods quantified from the Observation phase are expressed in hours (lower panel), with the standard deviation (Std.) for experimental groups shown in the upper panel. **C** The circadian power of four representative subjects from each group is presented in a spectrogram (**a**). The circadian power ratios (lower panel) and their standard deviations for each group (upper panel) in (**a**) are compared in (**b**). **D** mRNA expression of *Per2*, *Bmal1*, and *Rev-erbα* in SCN in each experimental group (*n* = 3 at each time point). Gray shading indicates darkness. Comparisons between groups at a given time point (*p* < *0.05*), namely, **a** between the control and POI groups, **b** between the CM and POI groups, **c** between the E2-CM and POI groups, and **d** between the CM and E-CM groups. Two-way ANOVA with Tukey’s test. Light and dark backgrounds represent light and dark phases of the day. ZT0 indicates the time when the light is switched on, and ZT12 indicates the time when the light is switched off. **E** (**a**) Localization analysis of PER2 protein expression in ovarian tissues accompanied by (**b**) corresponding serum estradiol concentrations of each experimental group (ng/ml, mean ± SD) were shown. Results obtained through immunohistochemical staining (*n* = 5) and estradiol ELISA assay (*n* = 15)

We hypothesized that the interplay between circadian rhythm disruptions and the components contributing to POI can be reflected in circadian locomotor rhythms. The disruptive effect of CTX and the repairing effects of CM and E2-CM were studied by examining the locomotor activity in POI mice. The experimental design is detailed in Fig. 5A, and circadian rhythms were quantified based on behavioral activities under constant darkness following a phase of treatment, as shown in Fig. 5B (‘Observ.’). As expected from the disruption at the molecular level, the CTX challenge in the POI group caused circadian rhythm heterogeneity, with highly variable circadian periods (Fig. 5B) and a decrease in circadian spectral power compared to other rhythmic powers (circadian power ratio, CPR; Fig. 5C). The circadian power indicates the dominance of the circadian rhythm among all rhythmic components of activities. While the circadian power tends to decrease after the CTX challenge (POI), this trend is not statistically significant due to increased variation. The variation, quantified by the standard deviation (‘Std.’), shows a clear trend: it is high in the POI group and normalizes to levels comparable to the control group in the CM and E2-CM treatment groups. The CM and E2-CM treatment effectively and consistently repaired the heterogeneity caused by CTX (Fig. 5B and C). Similar to results presented in Fig. 4, the gene expression related to the SCN such as *Per2*, *Bmal1*, and *Rev-erbα* were observed in these mice. As shown in Fig. 5D reveals that relative to the control group, the POI group showed a persistent increase in *Per2* expression and comparable changes in *Bmal1* and *Rev-erbα* expression (Fig. 5D, red line vs. blue line). Treatment with CM and E2-CM effectively rescued the CTX-induced expression defects of all three genes in the SCN of the POI mice (Fig. 5D, green and purple lines vs. red line). Notably, E2-CM was more effective than CM alone in rescuing the aforementioned CTX-induced expression defects. The expression of PER2 protein in the ovary tissues (Fig. 5E, panel a) and the serum E2 levels in each experimental groups (Fig. 5E, panel b) were further confirmed through immunohistochemical staining and ELISA assay, and the results clarified the rescue effects (Fig. 5E). Collectively, the aforementioned results clearly indicate the restorative effects of CM and E2-CM on CTX-induced circadian rhythm disturbances in POI mice.

### CM and E2-CM promoted angiogenesis in vitro and in vivo*,* with possible angiogenin involvement

**Fig. 6:**
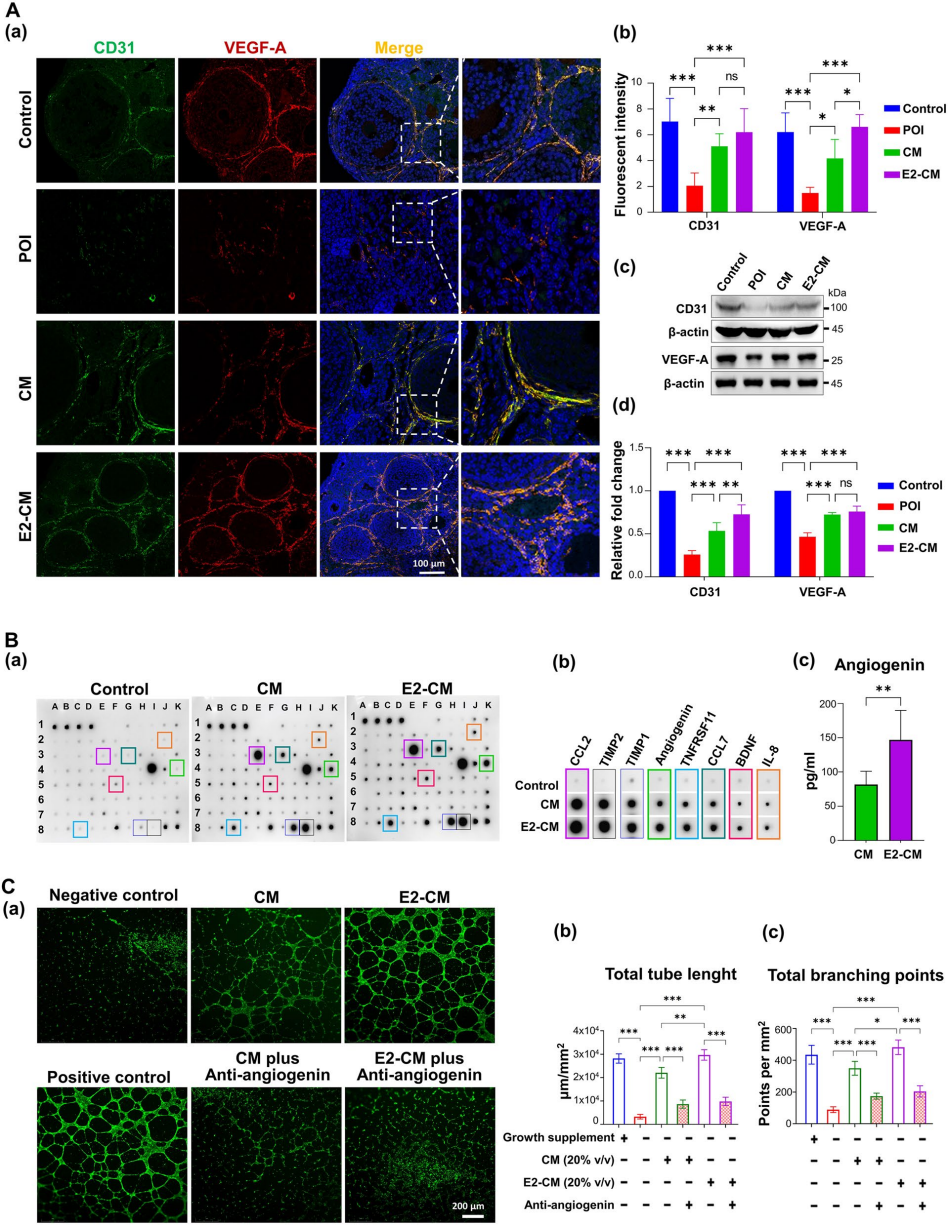
CM and E2-CM promoted angiogenesis in vitro and in vivo, possibly with angiogenin involvement.** A** Effects of CM/E2-CM on restoring CTX-affected ovarian angiogenesis in mice. Confocal images of IF staining (**a**) and quantitative analysis (*n* = 5) (**b**) for angiogenic marker CD31 (in green) and VEGF-A (in red) in ovary tissues of each experimental group. Immunoblotting results for CD31 and VEGF-A (**c**) and their and quantitative analysis (*n* = 3) (**d**). **B** Protein component analysis of CM and E2-CM. Results obtained through cytokine array analysis. Nonconcentrated medium pool from pcMSC passages 8–11. Total protein, 700 µg/ml. (**a**). Top eight abundant cytokines (**b**).). The concentration of Angiogenin in CM and E2-CM was shown. ELISA, **p < 0.01, Student *t* test (*n* = 8) (**c**). **C** Effects of CM and E2-CM on angiogenesis, as revealed through tube formation assay using human HUVEC cells. Fluorescent images depicting tube formation labeled with Calcein AM in experimental groups. Anti-angiogenin antibody used to neutralize angiogenin (**a**). Quantitation and statistical analysis of total tube length (**b**) and branching point number (**c**) under various experimental condition (*n* = *5*). *p < 0.05, **p < 0.01, ***p < 0.001. One-way ANOVA with Tukey’s test

Neovascularization plays a key role in ovarian hormone production and folliculogenesis. To investigate the potential role of CM and E2-CM in restoring CTX-induced angiogenesis defect in vivo, the protein levels of CD31 (a marker for vascular differentiation) and VEGF-A in ovarian tissues subjected to different experimental conditions were analyzed. We discovered that CTX treatment considerably reduced the protein levels of CD31 and VEGF-A in the ovarian tissues of the experimental mice, and that CM and E2-CM treatments significantly restored the CTX-induced defects (Fig. 6A, Panels a and b). The protein levels of VEGF-A and CD31 in the ovarian tissues of the experimental mice were further confirmed through Western blotting. Consistent with earlier observations, the changes in the E2-CM group were significantly more favorable than those in the CM group (Fig. 6A, Panels c and d).

To explore the potential mechanisms underlying the aforementioned findings, cytokine array analysis was performed to identify the cytokines of CM and E2-CM that might be involved in angiogenesis (angiogenin and TNFRSF11), extracellular matrix regulation (TIMP1 and TIMP2), immunoregulation (CCL2 and CCL7), and cell growth (IL-8 and IGFBP2) (Fig. 6B, Panels a and b). A basal medium without ER^+^pcMSC cultivation was used as a negative control group. We discovered that the total amount of secreted protein was higher in the E2-CM group than that in CM group, the individual expression levels are displayed for each group are presented as supplementary information (Fig. S4). Among the secreted protein levels, angiogenin was significantly higher in the E2-CM group compared to the CM group (Fig. 6B, Panel b: cytokine array; Panel c: ELISA assay).

To investigate whether angiogenin plays a role in CM/E2-CM-induced angiogenesis, HUVEC cells were treated with CM or E2-CM supplemented with or without angiogenin-neutralizing antibodies. Compared with the negative control (basal medium only), the CM and E2-CM groups exhibited the same effects experienced by the positive control with respect to the promotion of HUVEC tube formation. Neutralizing angiogenin in CM and E2-CM significantly suppressed the effects of CM and E2-CM on angiogenesis with respect to total tube length and total branch points (Fig. 6C). These results clearly highlight the role of angiogenin in the effects of CM and E2-CM on angiogenesis, which influences folliculogenesis and overall ovarian function.

### Exosomal miRNAs may influence effects of CM and E2-CM on CTX-induced POI and circadian rhythm regulation

**Fig. 7:**
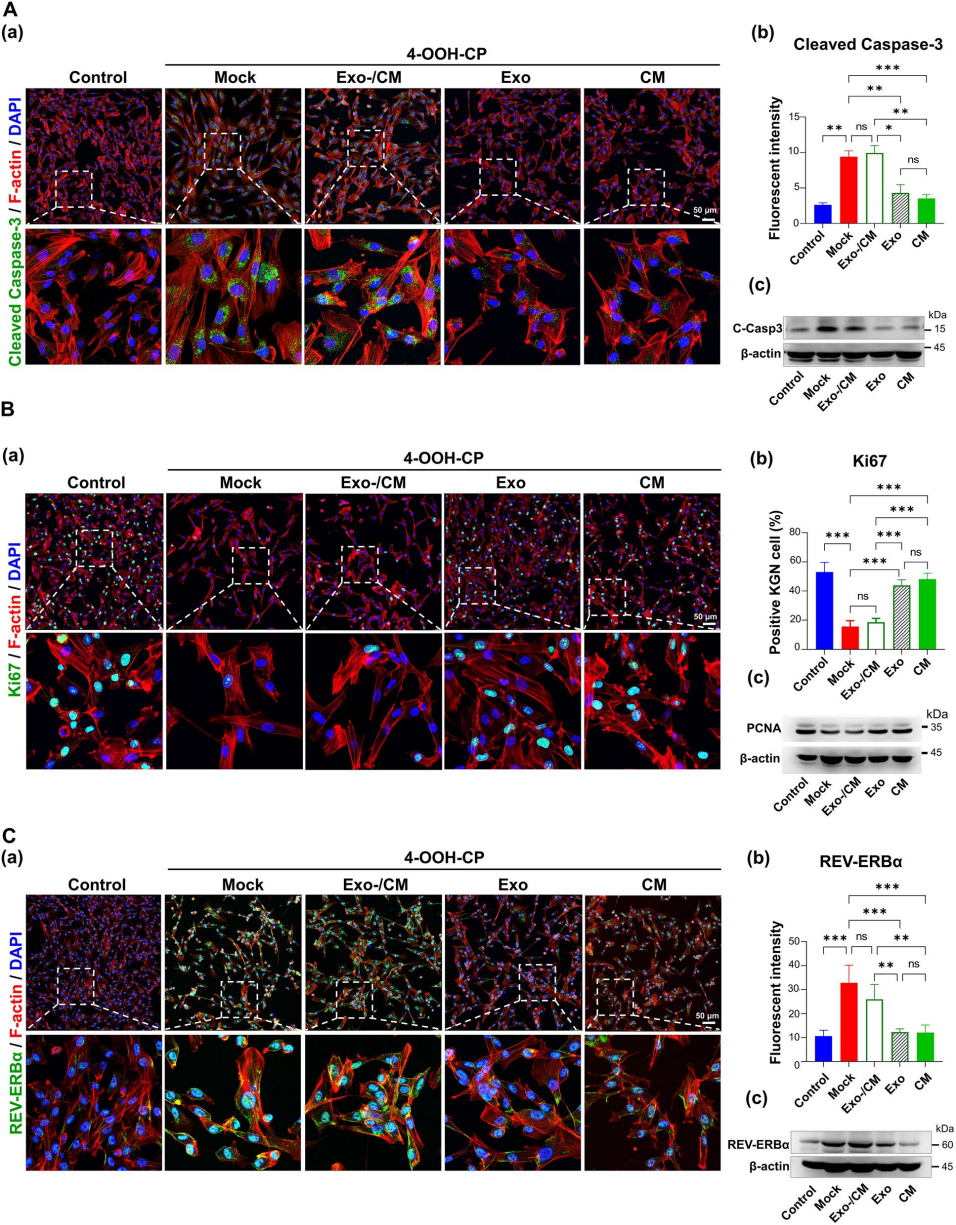
ER^+^pcMSC-derived exosomes effectively restore the defects induced by 4-OOH-CP (8 μg/ml for 12 h) in the KGN cell model. The experimental groups included the control group (Control group), the groups challenged with 4-OOH-CP then treated with: MDCD201 mock medium only (Mock group), exosome-depleted conditioned medium (Exo-/CM group), exosomes only (Exo group), and conditioned medium (CM group). **A** The effects of the various experimental groups on the protein expression of cleaved caspase-3 were shown. Immunofluorescence staining and quantitative analysis (**a**, **b**; *n* = 4) were performed, along with immunoblotting (**c**; *n* = 4). **B** The protein expressions of Ki67 were shown. Immunofluorescence staining and quantitative analysis **(a**, **b**; *n* = 4) were conducted, along with immunoblotting for PCNA (**c**; *n* = 4). **C** The protein expression levels of REV-ERBα in various experimental groups were shown. Immunofluorescence staining and quantitative analysis (**a**, **b**; *n* = 4) were performed, along with immunoblotting for PCNA (**c**; *n* = 4). *p < 0.05, **p < 0.01, ***p < 0.001. One-way ANOVA with Tukey’s test

The key role of exosomes in CM in regulating CTX-induced POI and circadian rhythm disorders was further investigated using a 4-OOH-CP-treated human KGN granulosa cell model in vitro. The experimental groups included the control group (Control group), the groups challenged with 4-OOH-CP then treated with: MDCD201 mock medium only (Mock group), exosome-depleted conditioned medium (Exo-/CM group), exosomes only (Exo group), and conditioned medium (CM group). We assessed the protein levels of C-Casp3 and PCNA (indicators of granulosa cell apoptosis and proliferation, Fig. 7A and B), as well as Rev-erbα and PER2 (related to circadian rhythm, Fig. 7C and Supplementary Fig. S5), using immunofluorescence staining and western blot analysis. Our findings indicate that the CM and Exosomes only groups significantly mitigated the effects of 4-OOH-CP (Mock group), while the Exosome-depleted CM (Exo-) group exhibited negative effects. These results underscore the critical role of the exosomal factors in CTX-induced POI and circadian rhythm disruption.

**Fig. 8:**
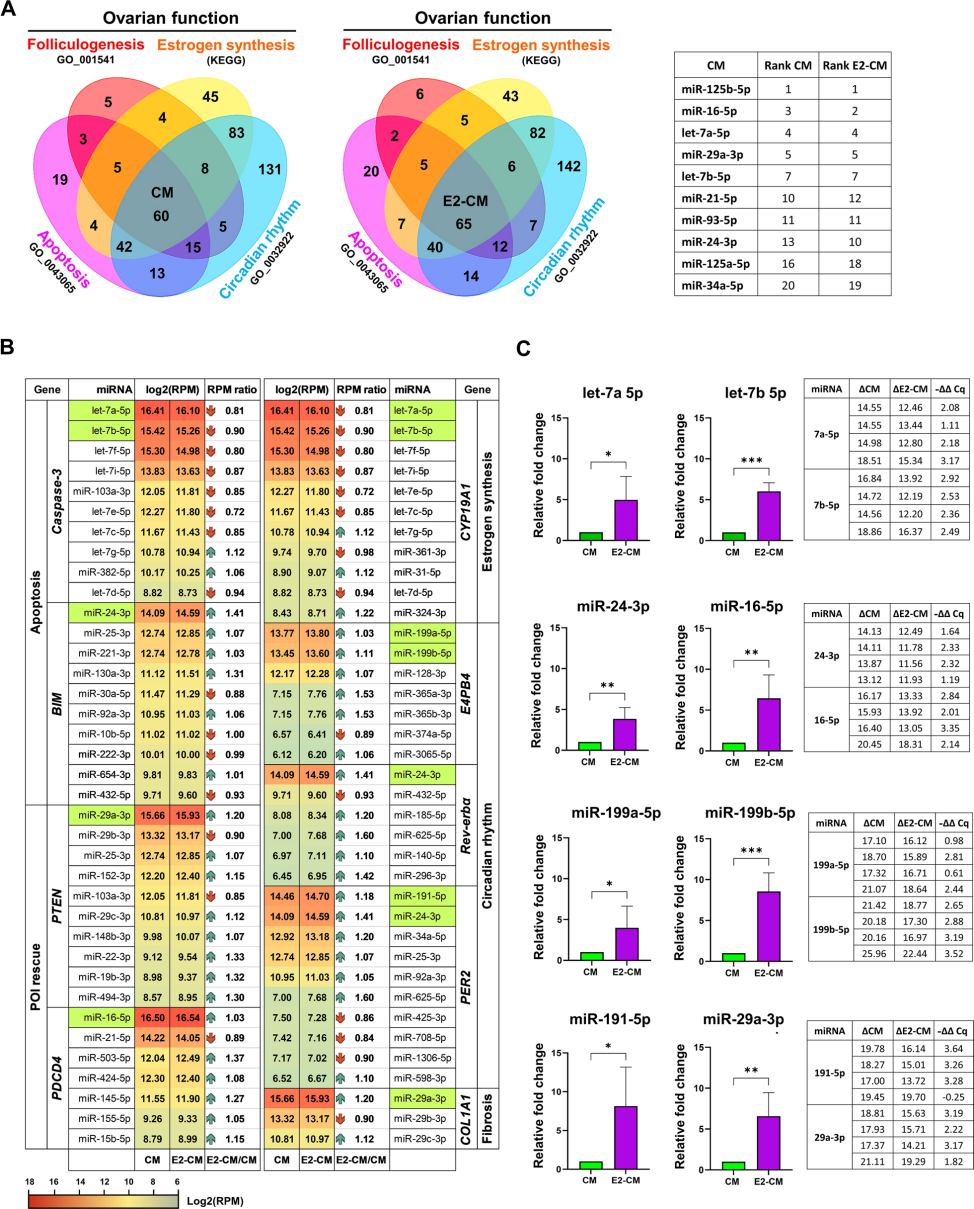
pcMSC Exosomal miRNA Profile. **A** Venn diagrams illustrates the overlap between pcMSC exosomal miRNAs from conditioned media (CM) on the left and E2-primed conditioned media (E2-CM) on the right, with gene sets related to ovarian follicle development (GO_0001541), estrogen synthesis (KEGG), apoptosis (GO_0043065), and circadian rhythm regulation (GO_0032922). The top miRNAs in the overlapping regions of these four sets are listed in the right panel. **B** Predicted interactions between miRNAs and genes of interest were presented in a heat map. TargetScanHuman 8.0 was used to calculate the targetability score, with only the top miRNAs having a TargetScore context +  + score of ≤ -0.2 selected. Genes of interest are categorized into groups such as apoptosis (*Caspase-3*, *BIM*), POI rescue (*PDCD4*, *PTEN*), estrogen synthesis (*CYP19A1*), circadian rhythm (*E4BP4*, *REV-ERBα*, *PER2*), and fibrosis (*COL1A1*). The expression of miRNAs is expressed as log2 RPM (Reads Per Million). RPM ratio columns depict the changes in miRNA expression after E2 priming. **C** Top miRNAs from each group were selected for qPCR analysis using miRNA-specific stem-loop RT primers. The relative fold changes in miRNA expression after estradiol priming are plotted using the 2^−ΔΔCq^ method. Absolute ΔCq and -ΔΔCq values are shown in the right panel. Total RNA samples were isolated from exosomes across passages 6 to 9 (*n* = 4). Student's *t*-test was performed; *p < 0.05, **p < 0.01

To further identify the potential molecular exosomal factors responsible for the therapeutic efficacy, the exosomal miRNAs present in CM and E2-CM were analyzed. As shown in Supplementary Figure S6, the E2-CM group showed a significant higher exosome counts then the CM group (Fig. S6A). The overall miRNA profiles in CM and E2-CM showed that there are miRNA candidates associated with apoptosis, POI, estrogen production, circadian rhythm modulation, and estrogen-circadian rhythm interactions (Figs. S6B–F). Figure 8A presented the key miRNA candidates associated with ovarian function (folliculogenesis GO_001541 and estrogen synthesis (KEGG), apoptosis (GO_0043065), and circadian rhythm (GO_0032922), and in both CM and E2-CM. Gene Set Enrichment Analysis (GSEA) of the four gene sets, which show statistically significant concordance with the corresponding four phenotypes of interest (as used in Fig. 8A), is presented in Fig. S7. This investigation was accomplished using the miRDB version 6 database (http://mirdb.org/, [49]), and miRNA targets sourced from miRDB were identified in MirTarget V4 (https://github.com/kassambara/miRTarget, [48]). Key targeted genes, such as those involved in apoptosis (*Caspase-3*, *BIM*), POI mitigation (*PDCD4* and *PTEN*) [65, 66], estrogen synthesis (*CYP19A1),* and ovarian circadian genes (*E4BP4, REV-ERBα, PER2*), fibrosis (*COL1A1*) (Fig. 8B) were targeted along with their corresponding and highly expressed miRNAs, which were identified using TargetScan 8.0 (https://www.targetscan.org/vert_80/ [50, 51]). We included the mRNAs with a TargetScan context +  + score ≤ − 0.2 for the general target analysis (Fig. 8B). We observed that pcMSC-derived exosomal miRNAs were involved in anti-apoptosis (let-7a-5p, let-7b-5p, let-7f-5p, and miR-103a-3p for inhibition of *Caspase-3*, miR-24-3p and miR-25-3p for targeting *BIM*), POI rescue (miR-29a-3p, and miR-29b-3p for *PTEN* and miR-16-5p, miR-21-5p for *PDCD4*), estrogen synthesis (let-7a-5p, let-7b-5p, let-7f-5p, let-7i-5p), and ovarian clock regulation (miR-199a-5p, miR-199b-5p, and miR-128-3p for *E4bp4;* miR-24-3p, miR-432-5p for *Rev-erbα*, miR-191-5p, miR-24-3p for *PER2*). In addition, there was also the expression of fibrosis-related miRNAs such as miR-29a-3p, miR-29b-3p for *Collagen I* (*COL1A1*).

The results pertaining to the interactions between miRNAs and their target genes are presented in Table 1, where only predominant miRNAs are highlighted. The profiling of exosomal miRNAs revealed a considerable secretion of miRNAs associated with cell longevity and circadian rhythm modulation. Notably, some miRNAs may simultaneously control two or three target genes such as miR-103a-3p (*Caspase-3*, *PTEN*), miR-24-3p (*PER2*, *REV-ERBα*, and *BIM*), miR-25-3p (*PTEN*, *PER2* and *BIM*), miR-107 (*Caspase-3*, *PTEN*). Furthermore, many of the let-7 family members are capable of targeting both *Caspase-3* and *CYP19A1*. (Fig. 8B).

**Table 1:**
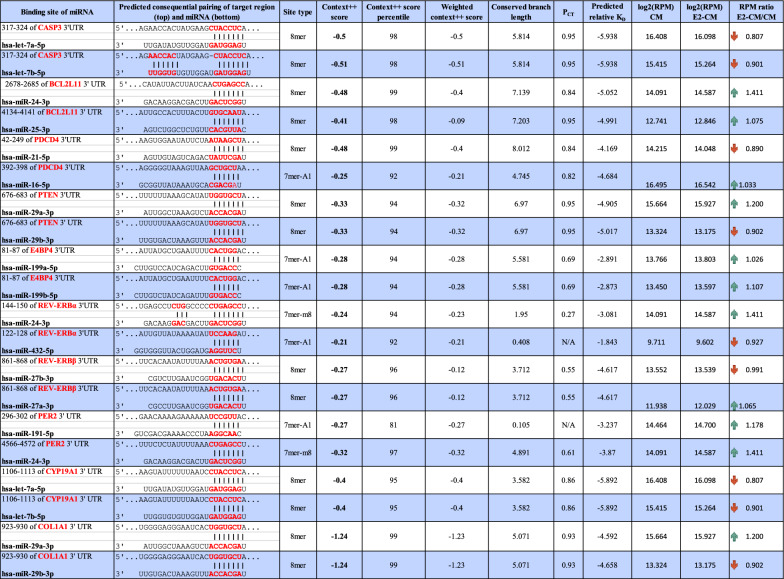
Putative interactions between miRNAs and their targets

For each target gene of interest, only the top two most abundant miRNAs with a TargetScan Context++ score of ≤ – 0.2 are included. The data in the table were sourced from TargetScan 8.0, and gene names, along with the pairing sequences between target genes and miRNAs, are highlighted in red

The expression levels of potential miRNA candidates in each group were further quantified using Q-RT PCR analysis. We found that E2 priming enhances the expression of miRNAs of ER^+^pcMSCs that regulate POI rescue and circadian rhythm. As shown in Fig. 8C, the levels of potential miRNA candidates were significantly higher in the E2-CM group compared to the CM group, including let-7a/b-5p (involved in apoptosis and estrogen synthesis), miR-24-3p (related to apoptosis and circadian rhythm), miR-16-5p (associated with POI rescue), miR-199a/b-5p, miR-191-5p (linked to circadian rhythm), and miR-29a-3p (related to POI rescue and fibrosis) (Fig. 8C).

The unique biosignature of ER-expressing MSCs in repairing POI and circadian rhythm disruption was further demonstrated by comparing the exosomal miRNA profiles of ER-positive MSCs (pcMSCs) with those of ER-negative MSCs (BMMSCs, ADMSCs, and WJ-MSCs (Data were referenced from Gene Expression Omnibus (GEO): GSE153752 (*n* = 4), GSE153752 (*n* = 4), GSE153752 (*n* = 4) correspond to BM, AD and WJ.), and the miRNAs targeting apoptosis (*Caspase 3, BIM*), POI rescue (*PTEN*, *PDCD4*), estrogen synthesis (*CYP11A1*), circadian rhythm (*PER2*, *Rev-erbα*, *E4BP4*), and fibrosis (*COL1A1*) are illustrated. The significant differences in miRNA expression profiles, especially targeting on the POI and circadian rhythm disruption, were observed between ER-positive pcMSCs and ER-negative MSCs (BMMSCs, ADMSCs, and UC-MSCs) (Fig. S8).

## Discussion

The present study is the first to demonstrate the therapeutic potential of the secretome derived from niche-responsive estrogen receptor-positive (ER +) human placenta–derived MSCs (ER^+^pcMSCs) in restoring both the CTX chemotherapy-induced–POI and circadian rhythm disorder in vivo and in vitro. It focused on the potential growth factor angiogenin and exosomal miRNAs in ER^+^pcMSC secretome (CM and E2-CM). The findings of this study can aid with the development of niche-responsive MSC cellular therapy for chemotherapy-induced POI and the circadian rhythm disruption.

The potential therapeutic effects of MSCs on POI have been reported in both preclinical studies and clinical trials. In addition to MSCs, CM obtained from various MSC types have been shown to effectively restore ovarian function in chemical-induced POI through various mechanisms. For example, CM derived from human umbilical cord MSCs has been demonstrated to improve the ovarian reserve of the premenopausal ovary through paracrine growth factors such as HGF, VEGF, and IGF-1 [28]; such CM has also been reported to prevent cisplatin-induced ovotoxicity through the G-CSF/PI3K/Akt signaling pathway. CM from human menstrual blood MSCs that contain high levels of FGF2 have been demonstrated to reduce tissue fibrosis and promote follicular growth [29]. Furthermore, CM derived from bone marrow MSCs has been reported to increase the expression of steroidogenesis-related genes, such as aromatase (CYP19A1) and StAR, which causes an increase in AMH and estrogen production in human granulosa cells [30]. CM can also promote angiogenesis in human ovarian microvascular endothelial cells by upregulating the PI3K/ALK pathway and increasing the expression of VEGF receptor 2, Tie2/Tek, VE-cadherin, endoglin, and VEGF [67]. However, no reports have considered the ovarian niche in demonstrating the therapeutic effect of MSCs or their secretome on POI-associated circadian rhythm disruption. In the present study, CM and E2-CM derived from ER^+^pcMSCs, which can respond to the ovarian estrogen niche, were demonstrated to effectively restore CTX-induced POI and circadian disruption.

We found that CTX led to significant alterations in ovary size, morphology, and function, including poor folliculogenesis and low E2 levels. Specifically, pcMSCs expressed ERα to respond to the estrogen in the ovarian niche. Treatment with CM and E2-CM derived from ER^+^pcMSCs significantly improved ovarian morphology and function in CTX-induced POI mice by restoring folliculogenesis and steroidogenesis and enhancing granulosa cell viability; this was achieved by promoting cell proliferation and reducing cell apoptosis. Crucially, our data also demonstrated that the expression of molecular clock genes is potentially involved in CTX-induced POI pathogenesis and the estrogen synthesis is directly controlled by the circadian clock through transcriptional elements. The CM and E2-CM derived from ER^+^pcMSCs not only rescued the E2 levels and ovarian folliculogenesis but also corrected disruptions in the circadian rhythm in this study’s CTX-induced POI mouse model (Figs. 1, 2, 4 and 5).

Studies have reported that *Rora* is crucial in the aromatase (CYP19A1) pathway for E2 synthesis [61, 68, 69]. The expression of *Cyp19a1* is regulated by the REV-ERB*α* /RORA loop, where REV-ERB*α* acts as an inhibitor, whereas RORA has an opposing effect [60, 61, 68, 69]. Additionally, the cholesterol side-chain cleavage enzyme encoded by *Cyp11a1* initiates estrogen synthesis and is under the inhibitory control of *E4bp4* [64]. We demonstrated, at both the gene and protein levels, that CTX enhances the expression of factors that inhibit estrogen synthesis such as REV-ERB*α* and E4BP4, thereby suppressing the expression levels of both *Cyp19a1* (Fig. 4A, Loop 2) and *Cyp11a1* (Fig. 4A, Loop 3), and increasing that of *Per2* (Figs. 4C and 5E). This increase in *Per2* explains the heterogeneous periods in the CTX-induced POI group in the mouse model (Fig. 5B). Furthermore, CTX significantly reduced the expression of the circadian-related protein RORA in the POI group. Significant changes in the Rora-associated expression of E4bp4, Rev-erbα, and Dbp were also observed (Fig. 4D and F). These results clearly highlight the role of RORA in transcriptional regulation through RRE and D-box elements (Fig. 4A, Loop 2 and 3). The rhythmic disruption and decrease of Rora expression may explain the reduction of E2 in POI, which impairs transcriptional regulation of both RRE-CYP10A1 (Fig. 4A, Loop 2) and D-box-CYP11A1 (Fig. 4A, Loop 3).

We assessed the CYP19A1 and CYP11A1 protein levels in ovarian tissues at ZT8 and ZT4, and our results indicate that CM and E2-CM treatment effectively reversed the CTX-induced suppression of CYP19A1 and CYP11A1. Similarly, CM and E2-CM restored the CTX-induced suppression of RORA protein levels in ovarian tissues at ZT4. The expression patterns of REV-ERB*α* and E4BP4 proteins exhibited an inverse relationship to those of RORA and DBP (Fig. 4D and E). We identified enhanced expression of estrogen synthesis pathway inhibitors such as REV-ERB*α* (Fig. 4D, F, and G) and E4BP4 (Fig. 4E and H) in the POI group, which indicates that the effects of CTX on the ovary are mediated by a mechanism that disrupts the expression of clock-controlled genes. Such disruptions can lead to changes in the expression of downstream genes with respect to both changes in expression levels (*Cyp19a1*) and rhythmicity (*Rora, Cyp11a1*). These results demonstrate the potential therapeutic effects of pcMSC-CM and E2-CM on the ovarian clock in the CTX-induced POI mouse model.

The exosomal miRNAs in CM and E2-CM apparently play a key role in reversing the effects of CTX on ovarian function and circadian rhythm disruption. miRNAs have been regarded as potential targeted therapeutic drugs because of their ability to regulate mRNA expression. Researchers have used several miRNAs to treat POI in mouse models, specifically targeting the genes of primordial follicle activation (*Pten*), apoptosis (*Pdcd4*) [65, 66, 70], ROS (SIRT4) [71], and oxidative stress (p38-Mapk14) [72]. Some therapeutic potential of MSC exosomal miRNAs in treating POI have been reported. Both miR-29a-3p and miR-21-5p, which were abundantly expressed in our CM and E2-CM (Fig. 8A), have demonstrated significant efficacy in this context [73, 74]. Additionally, miR-29a-3p has been shown to inhibit apoptosis, promote proliferation in granulosa cell models, and preserve and restore ovarian follicular function in mice [73]. Meanwhile, miR-21-5p, which is downregulated in the peripheral blood of patients with autoimmune POI, has also proven effective. Administration of exosomes loaded with miR-21-5p successfully restored ovarian structure and function in an autoimmune POI mouse model by regulating the MSX1-mediated Notch signaling pathway [74].

In the present study, we demonstrated the effects of CM and E2-CM on CTX-induced apoptosis and Caspase-3 expression in granulosa cells in vivo (Fig. 2) and in vitro (Fig. 3). We also observed high expression levels of several Caspase-3-associated miRNAs (i.e., let-7 family [-7a-5p, -7b-5p, -7f-5p, -7i-5p] and miR-103a-3p) in pcMSC-derived exosomes. The let-7 family is also recognized for its role in inhibiting premature ovarian failure [75]. miR-24-3p has been shown to inhibit apoptosis through suppressing the expression of *BIM* [76, 77]. Abundant expression of miR-24-3p along with other *BIM*-related miRNAs such as miR-25-3p, miR-221-3p was also observed (Fig. 8B). Additionally, miRNAs involved in ovary rescue, such as *PTEN* (e.g., miR-29a-3p, miR-29b-3p, and miR-25-3p), and *PDCD4* (e.g., miR-16-5p, miR-21-5p, and miR-503-5p) were also detected (Fig. 8B). In the present study, we observed that one of the consequences of POI induced by ovarian toxins is fibrosis (Fig. 2C). This is also consistent with other POI models [78, 79], in which collagen I deposition has explained the pathogenesis. We observed that pcMSC exosomes harbor abundant miR-29 family that is widely known to inhibit fibrosis (Fig. 8B) [80, 81].

Female reproductive function is regulated by under the circadian rhythms of the hypothalamic–pituitary–gonadal (HPG) axis, which help maintain ovarian hormone secretion and a regular reproductive cycle [58]. Estrogen has been identified as a key hormone in ovarian function with findings indicating that disruptions to the circadian rhythm can adversely affect estrogen levels [19, 82]. Estrogen and molecular clock components interact to regulate gene expression, cell biology, and circadian rhythm during the estrous cycle [19, 83, 84]. Disruptions to the ovarian clock, or mismatches between the ovarian clock and the central circadian oscillator, can lead to the onset and progression of reproductive pathologies [57, 58].

Our study demonstrates the effects of CM and E2-CM on CTX-induced circadian rhythm disorder involving the clock proteins RORA, REV-ERBα, E4BP4, DBP (Fig. 4) and PER2 (Fig. 5). The miRNAs derived from pcMSC-CM exosomes were involved in regulating ovarian clock genes, including miR-199a-5p, miR-199b-5p, and miR-128-3p for *E4BP4*; miR-24-3p and miR-432-5p for *REV-ERBα*, miR-191-5p, miR-24-3p, and miR-34a-5p for *PER2*. Notably, several highly expressed miRNAs simultaneously target multiple genes: miR-24-3p targets both *PER2* and *REV-ERBα*, miR-25-3p targets *PER2* and *PTEN*, and miR-103a-3p targets *Caspase-3* and *PTEN*. Additionally, miR-625-5p can simultaneously target both *PER2* and *REV-ERBα* (Fig. 8B).

The expression levels of certain miRNAs were notably elevated in both CM and E2-CM samples. Specifically, let-7a-5p, let-7b-5p, let-7i-5p, and miR-24-3p were associated with the regulation of apoptosis and estrogen synthesis, while miR-199a-5p, miR-199b-5p, miR-24-3p, and miR-191-5p were linked to the regulation of circadian rhythm. Additionally, miR-29a-3p and miR-16-5p played a role in follicle activation. These findings highlight the potential direction of miRNA therapy for POI and POI-related circadian rhythm disorder.

The estrogen responding ER^+^MSCs are ideal candidates in treating CTX-induced POI and circadian rhythm disruption. We found that E2 priming enhances the expression of miRNAs that regulate POI rescue and circadian rhythm. This was evidenced by qPCR assays using miRNA-specific stem-loop RT primers to analyze the top eight miRNAs representing the target groups of interest. Notably, we observed E2 increased expression of miRNAs that suppress apoptosis (let-7a/b-5p and miR-24-3p), rescue POI (miR-16-5p and miR-29a-3p), regulate circadian rhythm (miR-24-3p, miR-191-5p, and miR-199a/b-5p), and exhibit anti-fibrotic properties (miR-29a-3p) (Fig. 8C).

The coordinated time-dependent action of the HPG axis has been reported to maintain physiological hormone secretion and reproductive function in accordance with the diurnal cycle. It is directly involved in the regulation of ovarian steroid-associated genes such as *StAR*, *Cyp19a1*, *Cyp17a1*, and *Cyp11a1* [58]. The reflection of ovarian function manifests through the expression of genes associated with steroid synthesis, exemplified by aromatase (Cyp19a1), wherein the secretion of estradiol (E2) undergoes diurnal fluctuations [59]. Estradiol has been reported to shorten the circadian period [85], while CTX is known to prolong it [86]. Although hypothalamic oscillators have been widely reported to regulate the timing of reproductive biology, our data demonstrate that CM and E2-CM from ER^+^pcMSCs can repair the effects of CTX on the clock genes associated with the SCN and peripheral ovary tissues in POI mice. However, the role of peripheral oscillators in ovarian function requires further investigation. Therefore, the stem cell therapy implemented in this study could potentially restore ovarian estrogen production, thereby reestablishing balance in the circadian rhythm when disrupted by CTX, and consequently mitigating the adverse effects of CTX on ovarian function.

## Conclusion

**Fig. 9:**
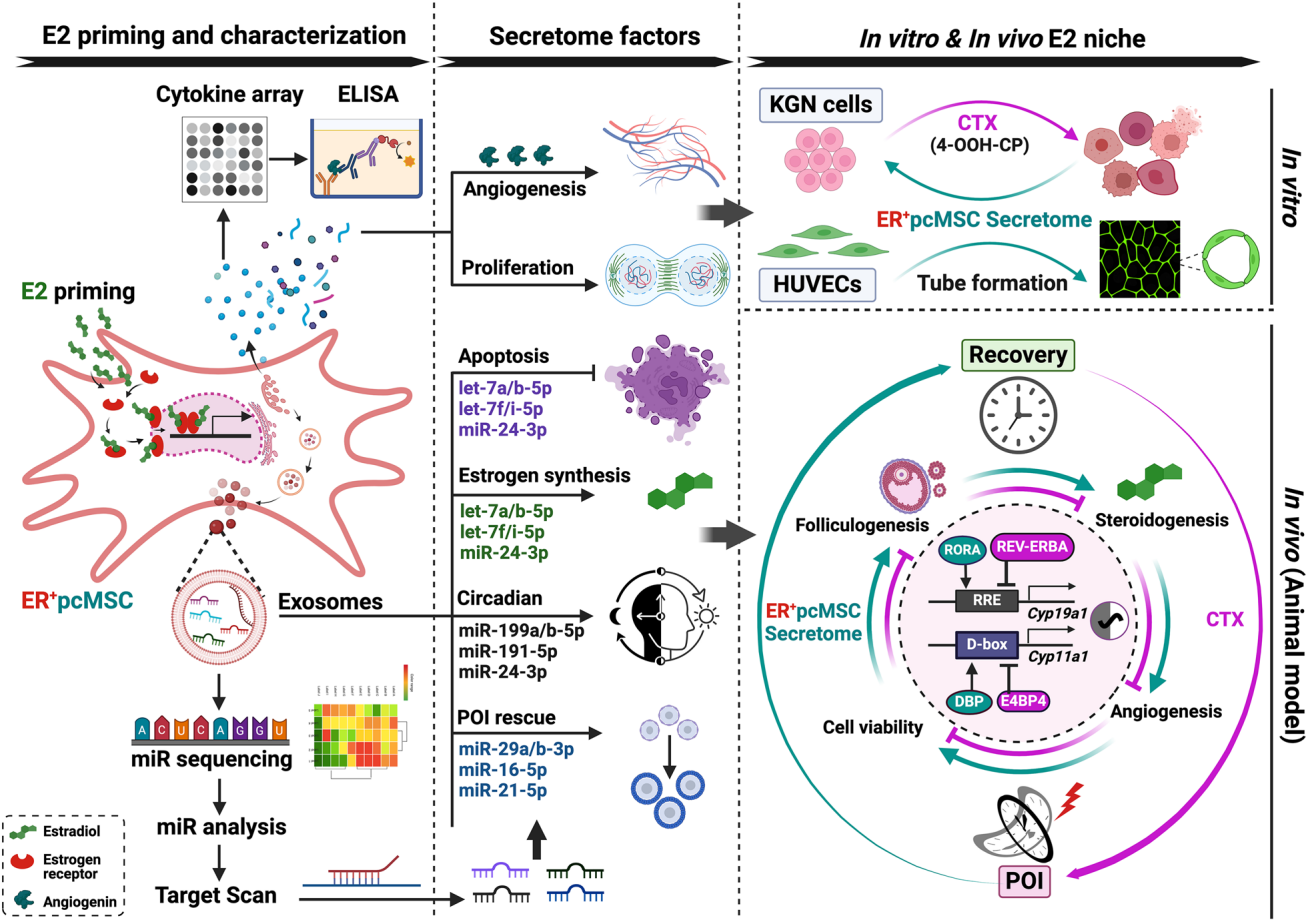
Proposed working model: The ER^+^pcMSC secretome restores CTX-induced POI and circadian rhythm disorder in vitro and in vivo*.* E2 priming and characterization of ER^+^pcMSC-derived secretome (left column); potential secreome factors, including cytokines and growth factors and exosomal miRNAs (middle column); and in vitro KGN cell model and in vivo mouse animal model (right column) revealing potential underlying mechanisms for restoring CTX-induced POI and circadian rhythm disruption

The present study is the first to demonstrate that the pcMSC secretome (with or without E2 priming) can respond to the ovarian niche and effectively restore ovarian failure and circadian rhythm disruption both in vitro and in vivo. The underlying mechanism in the present study’s CTX-induced POI mouse model involved angiogenin-related angiogenesis and granulosa cell viability*.* Notably, our study shows that CM and E2-CM from ER^+^pcMSCs can repair CTX-induced damage to the expression of estrogen synthesis-related genes, including *Cyp19a1, Cyp11a1*, and the ovarian clock-controlled genes such as *Rora, Rev-erbα, E4bp4*, and *Dbp* (Fig. 9). The findings from the present study offer a valuable reference point for the development of stem cellular therapy aimed at achieving ovarian regeneration and diurnal rhythm improvement in patients with chemotherapy-induced POI.

## Supplementary Information

Supplementary Material 1. Figure S1. Establishment of CTX-induced POI mouse model.

Supplementary Material 2. Figure S2. Expressions of estrogen receptorin pcMSCs

Supplementary Material 3. Figure S3. Effect of 4-OOH-CP on KGN cell viability. Related to Fig. 3

Supplementary Material 4. Figure S4. Generation and characterization of conditioned medium derived from ER + pcMSCs without or with E2 priming. Related to Fig. 6

Supplementary Material 5. Figure S5. Effect of 4OOH-CP on PER2 expression in KGN cells

Supplementary Material 6. Figure S6. Quantification and characterization of exosomes in conditioned medium derived from ER + pcMSCs without or with E2 priming. Related to Fig. 8

Supplementary Material 7. Figure S7. Gene Set Enrichment AnalysisRelated to Fig. 8

Supplementary Material 8. Figure S8. Exosomal miRNA expression in different MSC types

Supplementary Material 9. Table S1. Real-time PCR primers and product size. Table S2. List of antibodies. Table S3. qPCR miRNA-specific stem loop-RT primers

Supplementary Material 10.

## Data Availability

The small RNA sequencing data generated and analyzed during the current study are available in the Gene Expression Omnibus (GEO) repository, under accession number [GSE247568]. Additional data and materials supporting the findings of this study are available from the corresponding author upon reasonable request.

## Abbreviations

AMH: Anti-Müllerian hormone
ANG: Angiogenin
BCL2: B-cell lymphoma 2
BDNF: Brain–derived neurotrophic factor
BIM (Bcl-2-like protein 11): Bcl-2 Interacting Mediator of cell death
CASP3: Caspase-3
CCL2: C–C motif chemokine ligand 2
CCL7: C–C motif chemokine ligand 7
CM: Conditioned medium
CYP11A1: Cytochrome P450 family 11 subfamily A member 1
CYP19A1: Cytochrome P450 family 19 subfamily A member 1
Dbp: D-box binding PAR bZIP transcription factor
E4bp4 (Nfil3): Nuclear factor, interleukin 3 regulated
ER: Estrogen receptor
FGF: Fibroblast growth factor
FOXL2: Forkhead box L2
FSH: Follicle-stimulating hormone
HGF: Hepatocyte growth factor
HPG: Hypothalamic–pituitary–gonadal
HUVEC: Human umbilical vein endothelial cell
IGFBP2: Insulin-like growth factor binding protein 2
MSC: Mesenchymal stem cell
NAMPT: Nicotinamide phosphoribosyl transferase
pcMSC: Placenta choriodecidual membrane–derived mesenchymal stem cells
PCNA: Proliferating cell nuclear antigen
PDCD4: Programmed cell death 4
POF: Premature ovarian failure
POI: Primary ovarian insufficiency
PTEN: Phosphatase and tensin homolog
Rev-erbα (Nr1d1): Nuclear receptor subfamily 1 group D member 1
Rora: RAR-related orphan receptor A
ROS: Reactive oxygen species
SCN: Suprachiasmatic nucleus
SIRT4: Sirtuin 4
StAR: Steroidogenic acute regulatory protein
TIMP1: Metallopeptidase inhibitor 1
TIMP2: Metallopeptidase inhibitor 2
TNFRSF11: Tumor necrosis factor receptor superfamily member 11
TUNEL: Terminal deoxynucleotidyl transferase dUTP nick end labeling
VEGF-A: Vascular endothelial growth factor A

## References

[1] Webber L, Davies M, Anderson R, Bartlett J, Braat D, Cartwright B, Cifkova R, de Muinck Keizer-Schrama S, Hogervorst E, Janse F, Liao L, Vlaisavljevic V, Zillikens C, Vermeulen N, and European Society for Human Reproduction and Embryology (ESHRE) Guideline Group on POI. 10.1093/humrep/dew027 27008889

[2] Ishizuka B. Current understanding of the etiology, symptomatology, and treatment options in premature ovarian insufficiency (POI). Front Endocrinol (Lausanne). 2021. 10.3389/fendo.2021.626924. 33716979 10.3389/fendo.2021.626924 PMC7949002

[3] Kolp L. Premature ovarian failure. Diagnosis and management of ovarian disorders. 2nd ed. Elsevier; 2003. p. 169–80. 10.1016/B978-012053642-9/50018-1.

[4] Eckhardt S, Wellons M. Defining menopause: what is early, what is late? Primary ovarian insufficiency. Cham: Springer International Publishing; 2016. p. 1–17. 10.1007/978-3-319-22491-6_1.

[5] Committee opinion No. 698. Hormone therapy in primary ovarian insufficiency. Obstet Gynecol. 2017;129:e134–41. 10.1097/AOG.0000000000002044. 28426619 10.1097/AOG.0000000000002044

[6] Chen HF, Ho HN. Prospects of primary ovarian insufficiency patient-specific pluripotent stem cells for disease modeling and clinical impacts. Curr Women s Heal Rev. 2018. 10.2174/1573404813666170929121848.

[7] McDonald IR, Welt AADCK. Health-related quality of life in women with primary ovarian insufficiency: a scoping review of the literature and implications for targeted interventions. Hum Reprod. 2022;37:2817–30. 10.1093/humrep/deac200. 36102839 10.1093/humrep/deac200 PMC9989734

[8] Chon SJ, Umair Z, Yoon M-S. Premature ovarian insufficiency: past, present, and future. Front Cell Dev Biol. 2021. 10.3389/fcell.2021.672890. 34041247 10.3389/fcell.2021.672890 PMC8141617

[9] Committee opinion No. 605. Primary ovarian insufficiency in adolescents and young women. Obstet Gynecol. 2014;124:193–7. 10.1097/01.AOG.0000451757.51964.98. 24945456 10.1097/01.AOG.0000451757.51964.98

[10] Meirow D. Reproduction post-chemotherapy in young cancer patients. Mol Cell Endocrinol. 2000;169:123–31. 10.1016/S0303-7207(00)00365-8. 11155944 10.1016/s0303-7207(00)00365-8

[11] Giacomo M, Barchi M, Baudat F, Edelmann W, Keeney S, Jasin M. Distinct DNA-damage-dependent and -independent responses drive the loss of oocytes in recombination-defective mouse mutants. Proc Natl Acad Sci. 2005;102:737–42. 10.1073/pnas.0406212102. 15640358 10.1073/pnas.0406212102 PMC545532

[12] Jang H, Hong K, Choi Y. Melatonin and fertoprotective adjuvants: prevention against premature ovarian failure during chemotherapy. Int J Mol Sci. 2017;18:1221. 10.3390/ijms18061221. 28590419 10.3390/ijms18061221 PMC5486044

[13] Roness H, Gavish Z, Cohen Y, Meirow D. Ovarian follicle burnout: A universal phenomenon? Cell Cycle. 2013;12:3245–6. 10.4161/cc.26358. 24036538 10.4161/cc.26358 PMC3885633

[14] Bedoschi G, Navarro PA, Oktay K. Chemotherapy-induced damage to ovary: mechanisms and clinical impact. Future Oncol. 2016;12:2333–44. 10.2217/fon-2016-0176. 27402553 10.2217/fon-2016-0176 PMC5066134

[15] Emadi A, Jones RJ, Brodsky RA. Cyclophosphamide and cancer: golden anniversary. Nat Rev Clin Oncol. 2009;6:638–47. 10.1038/nrclinonc.2009.146. 19786984 10.1038/nrclinonc.2009.146

[16] Ganesan S, Madden JA, Keating AF. Ovarian metabolism of xenobiotics. In: Hoyer PB, editor. Ovarian toxicol. 2nd ed. CRC Press; 2013. p. 15–30. 10.1201/b15546.

[17] Robertson JA. Ethical issues in ovarian transplantation and donation. Fertil Steril. 2000;73:443–6. 10.1016/S0015-0282(99)00587-7. 10688993 10.1016/s0015-0282(99)00587-7

[18] Wilhoite MN, Warwar RE, Starostanko AN, Sax MR. Analysis of the literature and patient counseling considerations for planned oocyte cryopreservation. Obstet Gynecol. 2022;140:102–5. 10.1097/AOG.0000000000004825. 35849465 10.1097/AOG.0000000000004825

[19] Alvord VM, Kantra EJ, Pendergast JS. Estrogens and the circadian system. Semin Cell Dev Biol. 2022;126:56–65. 10.1016/j.semcdb.2021.04.010. 33975754 10.1016/j.semcdb.2021.04.010 PMC8573061

[20] Jiang Z, Zou K, Liu X, Gu H, Meng Y, Lin J, et al. Aging attenuates the ovarian circadian rhythm. J Assist Reprod Genet. 2021;38:33–40. 10.1007/s10815-020-01943-y. 32926298 10.1007/s10815-020-01943-y PMC7822988

[21] Yoshikawa T, Sellix M, Pezuk P, Menaker M. Timing of the ovarian circadian clock is regulated by gonadotropins. Endocrinology. 2009;150:4338–47. 10.1210/en.2008-1280. 19520783 10.1210/en.2008-1280 PMC2736075

[22] Anderson ST, FitzGerald GA. Sexual dimorphism in body clocks. Science. 1979;2020(369):1164–5. 10.1126/science.abd4964. 10.1126/science.abd4964 32883849

[23] Hatcher KM, Royston SE, Mahoney MM. Modulation of circadian rhythms through estrogen receptor signaling. Eur J Neurosci. 2020;51:217–28. 10.1111/ejn.14184. 30270552 10.1111/ejn.14184

[24] Li S, Wang M, Ao X, Chang AK, Yang C, Zhao F, et al. CLOCK is a substrate of SUMO and sumoylation of CLOCK upregulates the transcriptional activity of estrogen receptor-α. Oncogene. 2013;32:4883–91. 10.1038/onc.2012.518. 23160374 10.1038/onc.2012.518

[25] Gery S, Virk RK, Chumakov K, Yu A, Koeffler HP. The clock gene Per2 links the circadian system to the estrogen receptor. Oncogene. 2007;26:7916–20. 10.1038/sj.onc.1210585. 17599055 10.1038/sj.onc.1210585

[26] Liu Y, Johnson BP, Shen AL, Wallisser JA, Krentz KJ, Moran SM, et al. Loss of BMAL1 in ovarian steroidogenic cells results in implantation failure in female mice. Proc Natl Acad Sci. 2014;111:14295–300. 10.1073/pnas.1209249111. 25225411 10.1073/pnas.1209249111 PMC4191810

[27] Ratajczak CK, Boehle KL, Muglia LJ. Impaired steroidogenesis and implantation failure in *Bmal1-/-* mice. Endocrinology. 2009;150:1879–85. 10.1210/en.2008-1021. 19056819 10.1210/en.2008-1021 PMC5393263

[28] Li J, Mao Q, He J, She H, Zhang Z, Yin C. Human umbilical cord mesenchymal stem cells improve the reserve function of perimenopausal ovary via a paracrine mechanism. Stem Cell Res Ther. 2017;8:55. 10.1186/s13287-017-0514-5. 28279229 10.1186/s13287-017-0514-5 PMC5345137

[29] Abd-Allah SH, Shalaby SM, Pasha HF, El-Shal AS, Raafat N, Shabrawy SM, et al. Mechanistic action of mesenchymal stem cell injection in the treatment of chemically induced ovarian failure in rabbits. Cytotherapy. 2013;15:64–75. 10.1016/j.jcyt.2012.08.001. 23260087 10.1016/j.jcyt.2012.08.001

[30] Park H, Chugh RM, El Andaloussi A, Hobeika E, Esfandyari S, Elsharoud A, et al. Human BM-MSC secretome enhances human granulosa cell proliferation and steroidogenesis and restores ovarian function in primary ovarian insufficiency mouse model. Sci Rep. 2021;11:4525. 10.1038/s41598-021-84216-7. 33633319 10.1038/s41598-021-84216-7 PMC7907146

[31] Zhang S, Huang B, Su P, Chang Q, Li P, Song A, et al. Concentrated exosomes from menstrual blood-derived stromal cells improves ovarian activity in a rat model of premature ovarian insufficiency. Stem Cell Res Ther. 2021;12:178. 10.1186/s13287-021-02255-3. 33712079 10.1186/s13287-021-02255-3 PMC7953711

[32] Uhlén M, Fagerberg L, Hallström BM, Lindskog C, Oksvold P, Mardinoglu A, et al. Tissue-based map of the human proteome: ESR1 and ESR2. Science (1979). 2015. 10.1126/science.1260419. 10.1126/science.1260419 25613900

[33] Karlsson M, Zhang C, Méar L, Zhong W, Digre A, Katona B, et al. A single–cell type transcriptomics map of human tissues: ESR1 and ESR2. Sci Adv. 2021. 10.1126/sciadv.abh2169. 34321199 10.1126/sciadv.abh2169 PMC8318366

[34] Hong L, Colpan A, Peptan IA, Daw J, George A, Evans CA. 17-β estradiol enhances osteogenic and adipogenic differentiation of human adipose-derived stromal cells. Tissue Eng. 2007;13:1197–203. 10.1089/ten.2006.0317. 17518737 10.1089/ten.2006.0317

[35] Huang T, Lu Z, Wang Z, Cheng L, Gao L, Gao J, et al. Targeting adipocyte ESRRA promotes osteogenesis and vascular formation in adipocyte-rich bone marrow. Nat Commun. 2024;15:3769. 10.1038/s41467-024-48255-8. 38704393 10.1038/s41467-024-48255-8 PMC11069533

[36] Su L-J, Wu M-S, Hui YY, Chang B-M, Pan L, Hsu P-C, et al. Fluorescent nanodiamonds enable quantitative tracking of human mesenchymal stem cells in miniature pigs. Sci Rep. 2017;7:45607. 10.1038/srep45607. 28358111 10.1038/srep45607 PMC5372358

[37] Nishi Y, Yanase T, Mu Y-M, Oba K, Ichino I, Saito M, et al. Establishment and characterization of a steroidogenic human granulosa-like tumor cell line, KGN, that expresses functional follicle-stimulating hormone receptor. Endocrinology. 2001;142:437–45. 10.1210/endo.142.1.7862. 11145608 10.1210/endo.142.1.7862

[38] Li J, Yu Q, Huang H, Deng W, Cao X, Adu-Frimpong M, et al. Human chorionic plate-derived mesenchymal stem cells transplantation restores ovarian function in a chemotherapy-induced mouse model of premature ovarian failure. Stem Cell Res Ther. 2018;9:81. 10.1186/s13287-018-0819-z. 29615109 10.1186/s13287-018-0819-z PMC5883538

[39] Myung J, Hong S, DeWoskin D, De Schutter E, Forger DB, Takumi T. GABA-mediated repulsive coupling between circadian clock neurons in the SCN encodes seasonal time. Proc Natl Acad Sci. 2015. 10.1073/pnas.1421200112. 26130804 10.1073/pnas.1421200112 PMC4517217

[40] Myung J, Wu M-Y, Lee C-Y, Rahim AR, Truong VH, Wu D, et al. The kidney clock contributes to timekeeping by the master circadian clock. Int J Mol Sci. 2019;20:2765. 10.3390/ijms20112765. 31195684 10.3390/ijms20112765 PMC6600447

[41] Myung J, Hong S, Hatanaka F, Nakajima Y, De Schutter E, Takumi T. Period coding of Bmal1 oscillators in the suprachiasmatic nucleus. J Neurosci. 2012;32:8900–18. 10.1523/JNEUROSCI.5586-11.2012. 22745491 10.1523/JNEUROSCI.5586-11.2012 PMC6622328

[42] Truong VH, Myung J. LocoBox: Modular hardware and open-source software for circadian entrainment and behavioral monitoring in home cages. Sensors. 2023;23:9469. 10.3390/s23239469. 38067841 10.3390/s23239469 PMC10708669

[43] E F, Zhang H, Yin W, Wang C, Liu Y, Li Y, et al. CPEB3 deficiency in mice affect ovarian follicle development and causes premature ovarian insufficiency. Cell Death Dis. 2021;13:21. 10.1038/s41419-021-04374-4. 34930897 10.1038/s41419-021-04374-4 PMC8688431

[44] Bolger AM, Lohse M, Usadel B. Trimmomatic: a flexible trimmer for Illumina sequence data. Bioinformatics. 2014;30:2114–20. 10.1093/bioinformatics/btu170. 24695404 10.1093/bioinformatics/btu170 PMC4103590

[45] Friedländer MR, Mackowiak SD, Li N, Chen W, Rajewsky N. miRDeep2 accurately identifies known and hundreds of novel microRNA genes in seven animal clades. Nucleic Acids Res. 2012;40:37–52. 10.1093/nar/gkr688. 21911355 10.1093/nar/gkr688 PMC3245920

[46] Langmead B, Trapnell C, Pop M, Salzberg SL. Ultrafast and memory-efficient alignment of short DNA sequences to the human genome. Genome Biol. 2009;10:R25. 10.1186/gb-2009-10-3-r25. 19261174 10.1186/gb-2009-10-3-r25 PMC2690996

[47] Rastegari E, Kajal K, Tan B-S, Huang F, Chen R-H, Hsieh T-S, et al. WD40 protein Wuho controls germline homeostasis via TRIM-NHL tumor suppressor Mei-p26 in Drosophila. Development. 2020. 10.1242/dev.182063. 31941704 10.1242/dev.182063 PMC7375833

[48] Liu W, Wang X. Prediction of functional microRNA targets by integrative modeling of microRNA binding and target expression data. Genome Biol. 2019;20:18. 10.1186/s13059-019-1629-z. 30670076 10.1186/s13059-019-1629-z PMC6341724

[49] Chen Y, Wang X. miRDB: an online database for prediction of functional microRNA targets. Nucleic Acids Res. 2020;48:D127–31. 10.1093/nar/gkz757. 31504780 10.1093/nar/gkz757 PMC6943051

[50] McGeary SE, Lin KS, Shi CY, Pham TM, Bisaria N, Kelley GM, et al. The biochemical basis of microRNA targeting efficacy. Science. 1979;2019:366. 10.1126/science.aav1741. 10.1126/science.aav1741 PMC7051167 31806698

[51] Agarwal V, Bell GW, Nam J-W, Bartel DP. Predicting effective microRNA target sites in mammalian mRNAs. Elife. 2015. 10.7554/eLife.05005. 26267216 10.7554/eLife.05005 PMC4532895

[52] Czimmerer Z, Hulvely J, Simandi Z, Varallyay E, Havelda Z, Szabo E, et al. A versatile method to design stem-loop primer-based quantitative PCR assays for detecting small regulatory RNA molecules. PLoS One. 2013;8: e55168. 10.1371/journal.pone.0055168. 23383094 10.1371/journal.pone.0055168 PMC3561390

[53] Britt KL, Saunders PK, McPherson SJ, Misso ML, Simpson ER, Findlay JK. Estrogen actions on follicle formation and early follicle development. Biol Reprod. 2004;71:1712–23. 10.1095/biolreprod.104.028175. 15269096 10.1095/biolreprod.104.028175

[54] Chi H, Cao Z. Effect of oestrogen on mouse follicle growth and meiotic resumption. Zygote. 2022;30:330–7. 10.1017/S0967199421000708. 34704551 10.1017/S0967199421000708

[55] Chiang MD, Chang C-Y, Shih H-J, Le VL, Huang Y-H, Huang C-J. Exosomes from human placenta choriodecidual membrane-derived mesenchymal stem cells mitigate endoplasmic reticulum stress, inflammation, and lung injury in lipopolysaccharide-treated obese mice. Antioxidants. 2022;11:615. 10.3390/antiox11040615. 35453300 10.3390/antiox11040615 PMC9029526

[56] Chang C-Y, Chen K-Y, Shih H-J, Chiang M, Huang I-T, Huang Y-H, et al. Let-7i-5p mediates the therapeutic effects of exosomes from human placenta choriodecidual membrane-derived mesenchymal stem cells on mitigating endotoxin-induced mortality and liver injury in high-fat diet-induced obese mice. Pharmaceuticals. 2021;15:36. 10.3390/ph15010036. 35056093 10.3390/ph15010036 PMC8779189

[57] Sellix MT, Menaker M. Circadian clocks in the ovary. Trends Endocrinol Metab. 2010;21:628–36. 10.1016/j.tem.2010.06.002. 20599392 10.1016/j.tem.2010.06.002 PMC2949464

[58] Shao S, Zhao H, Lu Z, Lei X, Zhang Y. Circadian rhythms within the female HPG axis: from physiology to etiology. Endocrinology. 2021. 10.1210/endocr/bqab117. 34125877 10.1210/endocr/bqab117 PMC8256628

[59] Chu M, Sun Z, Xiang Y, et al. Leptin receptor mediates bmal1 regulation of estrogen synthesis in granulosa cells. Animals. 2019;9:899. 10.3390/ani9110899. 31683864 10.3390/ani9110899 PMC6912815

[60] Wang L, Li J, Zhang L, Shi S, Zhou X, Hu Y, et al. NR1D1 targeting CYP19A1 inhibits estrogen synthesis in ovarian granulosa cells. Theriogenology. 2022;180:17–29. 10.1016/j.theriogenology.2021.12.009. 34933195 10.1016/j.theriogenology.2021.12.009

[61] Yu H, Niu Y, Jia G, Liang Y, Chen B, Sun R, et al. Maternal diabetes-mediated RORA suppression in mice contributes to autism-like offspring through inhibition of aromatase. Commun Biol. 2022;5:51. 10.1038/s42003-022-03005-8. 35027651 10.1038/s42003-022-03005-8 PMC8758718

[62] Dierickx P, Zhu K, Carpenter BJ, Jiang C, Vermunt MW, Xiao Y, et al. Circadian REV-ERBs repress E4bp4 to activate NAMPT-dependent NAD+ biosynthesis and sustain cardiac function. Nat Cardiovasc Res. 2021;1:45–58. 10.1038/s44161-021-00001-9. 35036997 10.1038/s44161-021-00001-9 PMC8754391

[63] Cowell IG. E4BP4/NFIL3, a PAR-related bZIP factor with many roles. BioEssays. 2002;24:1023–9. 10.1002/bies.10176. 12386933 10.1002/bies.10176

[64] Mukherji A, Kobiita A, Ye T, Chambon P. Homeostasis in intestinal epithelium is orchestrated by the circadian clock and microbiota cues transduced by TLRs. Cell. 2013;153:812–27. 10.1016/j.cell.2013.04.020. 23663780 10.1016/j.cell.2013.04.020

[65] Fu X, He Y, Wang X, Peng D, Chen X, Li X, et al. Overexpression of miR-21 in stem cells improves ovarian structure and function in rats with chemotherapy-induced ovarian damage by targeting PDCD4 and PTEN to inhibit granulosa cell apoptosis. Stem Cell Res Ther. 2017;8:187. 10.1186/s13287-017-0641-z. 28807003 10.1186/s13287-017-0641-z PMC5556338

[66] Thabet E, Yusuf A, Abdelmonsif DA, Nabil I, Mourad G, Mehanna RA. Extracellular vesicles miRNA-21: a potential therapeutic tool in premature ovarian dysfunction. Mol Hum Reprod. 2020;26:906–19. 10.1093/molehr/gaaa068. 33049041 10.1093/molehr/gaaa068

[67] Park H-S, Ashour D, Elsharoud A, Chugh RM, Ismail N, EL Andaloussi A, et al. Towards cell free therapy of premature ovarian insufficiency: Human bone marrow mesenchymal stem cells secretome enhances angiogenesis in human ovarian microvascular endothelial cells. J Stem Cells Res Dev Ther. 2019;5:1–8. 10.24966/SRDT-2060/100019. 10.24966/srdt-2060/100019 PMC7269190 32494757

[68] Sarachana T, Hu VW. Differential recruitment of coregulators to the RORA promoter adds another layer of complexity to gene (dys) regulation by sex hormones in autism. Mol Autism. 2013;4:39. 10.1186/2040-2392-4-39. 24119295 10.1186/2040-2392-4-39 PMC4016566

[69] Sarachana T, Xu M, Wu R-C, Hu VW. Sex hormones in autism: Androgens and estrogens differentially and reciprocally regulate RORA, a novel candidate gene for autism. PLoS One. 2011;6: e17116. 10.1371/journal.pone.0017116. 21359227 10.1371/journal.pone.0017116 PMC3040206

[70] Yang M, Lin L, Sha C, Li T, Zhao D, Wei H, et al. Bone marrow mesenchymal stem cell-derived exosomal miR-144-5p improves rat ovarian function after chemotherapy-induced ovarian failure by targeting PTEN. Lab Investig. 2020;100:342–52. 10.1038/s41374-019-0321-y. 31537899 10.1038/s41374-019-0321-y

[71] Ding C, Qian C, Hou S, Lu J, Zou Q, Li H, et al. Exosomal miRNA-320a is released from hAMSCs and regulates SIRT4 to prevent reactive oxygen species generation in POI. Mol Ther Nucleic Acids. 2020;21:37–50. 10.1016/j.omtn.2020.05.013. 32506013 10.1016/j.omtn.2020.05.013 PMC7272510

[72] Liu T, Lin J, Chen C, Nie X, Dou F, Chen J, et al. MicroRNA-146b-5p overexpression attenuates premature ovarian failure in mice by inhibiting the Dab2ip/Ask1/p38-Mapk pathway and γH2A.X phosphorylation. Cell Prolif. 2021. 10.1111/cpr.12954. 33166004 10.1111/cpr.12954 PMC7791167

[73] Gao T, Cao Y, Hu M, Du Y. Human umbilical cord mesenchymal stem cell-derived extracellular vesicles carrying microRNA-29a improves ovarian function of mice with primary ovarian insufficiency by targeting HMG-Box transcription factor/Wnt/β-Catenin signaling. Dis Markers. 2022;2022:1–19. 10.1155/2022/5045873. 10.1155/2022/5045873 PMC9277157 35845134

[74] Yang Y, Tang L, Xiao Y, Huang W, Gao M, Xie J, et al. miR-21-5p-loaded bone mesenchymal stem cell-derived exosomes repair ovarian function in autoimmune premature ovarian insufficiency by targeting MSX1. Reprod Biomed Online. 2024;48: 103815. 10.1016/j.rbmo.2024.103815. 38582043 10.1016/j.rbmo.2024.103815

[75] Li Y, Fang Y, Liu Y, Yang X. MicroRNAs in ovarian function and disorders. J Ovarian Res. 2015;8:51. 10.1186/s13048-015-0162-2. 26232057 10.1186/s13048-015-0162-2 PMC4522283

[76] Soroosh A, Fang K, Hoffman JM, Law IKM, Videlock E, Lokhandwala ZA, et al. Loss of miR-24-3p promotes epithelial cell apoptosis and impairs the recovery from intestinal inflammation. Cell Death Dis. 2021;13:8. 10.1038/s41419-021-04463-4. 34923573 10.1038/s41419-021-04463-4 PMC8684555

[77] Nouws J, Wan F, Finnemore E, Roque W, Kim S-J, Bazan I, et al. MicroRNA miR-24-3p reduces DNA damage responses, apoptosis, and susceptibility to chronic obstructive pulmonary disease. JCI Insight. 2021. 10.1172/jci.insight.134218. 33290275 10.1172/jci.insight.134218 PMC7934877

[78] Cui L, Bao H, Liu Z, Man X, Liu H, Hou Y, et al. hUMSCs regulate the differentiation of ovarian stromal cells via TGF-β1/Smad3 signaling pathway to inhibit ovarian fibrosis to repair ovarian function in POI rats. Stem Cell Res Ther. 2020;11:386. 10.1186/s13287-020-01904-3. 32894203 10.1186/s13287-020-01904-3 PMC7487655

[79] Nazdikbin Yamchi N, Ahmadian S, Mobarak H, Amjadi F, Beheshti R, Tamadon A, et al. Amniotic fluid-derived exosomes attenuated fibrotic changes in POI rats through modulation of the TGF-β/Smads signaling pathway. J Ovarian Res. 2023;16:118. 10.1186/s13048-023-01214-1. 37370156 10.1186/s13048-023-01214-1 PMC10294370

[80] Cushing L, Kuang PP, Qian J, Shao F, Wu J, Little F, et al. miR-29 is a major regulator of genes associated with pulmonary fibrosis. Am J Respir Cell Mol Biol. 2011;45:287–94. 10.1165/rcmb.2010-0323OC. 20971881 10.1165/rcmb.2010-0323OC PMC3175558

[81] Chioccioli M, Roy S, Newell R, Pestano L, Dickinson B, Rigby K, et al. A lung targeted miR-29 mimic as a therapy for pulmonary fibrosis. EBioMedicine. 2022;85: 104304. 10.1016/j.ebiom.2022.104304. 36265417 10.1016/j.ebiom.2022.104304 PMC9587275

[82] Ball LJ, Palesh O, Kriegsfeld LJ. The pathophysiologic role of disrupted circadian and neuroendocrine rhythms in breast carcinogenesis. Endocr Rev. 2016;37:450–66. 10.1210/er.2015-1133. 27712099 10.1210/er.2015-1133 PMC5045494

[83] Rivera HM, Stincic TL. Estradiol and the control of feeding behavior. Steroids. 2018;133:44–52. 10.1016/j.steroids.2017.11.011. 29180290 10.1016/j.steroids.2017.11.011 PMC5864536

[84] Mauvais-Jarvis F, Clegg DJ, Hevener AL. The role of estrogens in control of energy balance and glucose homeostasis. Endocr Rev. 2013;34:309–38. 10.1210/er.2012-1055. 23460719 10.1210/er.2012-1055 PMC3660717

[85] Morin LP, Fitzgerald KM, Zucker I. Estradiol shortens the period of hamster circadian rhythms. Science. 1979;1977(196):305–7. 10.1126/science.557840. 10.1126/science.557840 557840

[86] Morgans LF, Burns R. Effect of cyclophosphamide on circadian rhythms in mitosis and DNA synthesis in normal mice and mice bearing the Ehrlich ascites carcinoma. Oncology. 1984;41:135–9. 10.1159/000225808. 6709276 10.1159/000225808

