# Supplementary material for: Secretome from estrogen-responding human placenta-derived mesenchymal stem cells rescues ovarian function and circadian rhythm in mice with cyclophosphamide-induced primary ovarian insufficiency
Source: J Biomed Sci. 2024 Oct 11;31:95. doi: 10.1186/s12929-024-01085-8 (PMC11468397; doi:10.1186/s12929-024-01085-8)
Supplement: Supplementary file 9 — Supplementary Material 9. Table S1. Real-time PCR primers and product size. Table S2. List of antibodies. Table S3. qPCR miRNA-specific stem loop-RT primers [file 12929_2024_1085_MOESM9_ESM.docx]

**Supplementary Tables**

**Table S1. Real-time PCR primers and product size**

| Gene | Species | Accession | Primer | Sequence | Product Size (bp) |
| --- | --- | --- | --- | --- | --- |
| *Actb* | Mouse | NM_007393 | Forward  Reverse | GCCCTGAGGCTCTTTTCCAG  TGCCACAGGATTCCATACCC | 51 |
| *Bmal1* | Mouse | NM_007489 | Forward  Reverse | GCAGTGCCACTGACTACCAAGA  TCCTGGACATTGCATTGCAT | 201 |
| *Cyp11a1* | Mouse | NM_019779.4 | Forward  Reverse | CCAGTGTCCCCATGCTCAAC  TGCATGGTCCTTCCAGGTCT | 74 |
| *Cyp19a1* | Mouse | NM_007810.4 | Forward  Reverse | CGGGCTACGTGGATGTGTT  GAGCTTGCCAGGCGTTAAAG | 135 |
| *Dbp* | Mouse | NM_016974.4 | Forward  Reverse | AATGACCTTTGAACCTGATCCCGCT  GCTCCAGTACTTCTCATCCTTCTGT | 175 |
| *E4bp4* | Mouse | NM_017373.3 | Forward  Reverse | CTCTTTCTCCACTTACTCCCAC  TTGTTCGTCTTCCCCATCAG | 127 |
| *Gapdh* | Mouse | NM_008084 | Forward  Reverse | AGGTCGGTGTGAACGGATTTG TGTAGACCATGTAGTTGAGGTCA | 129 |
| *Rev-erbα* | Mouse | NM_145434.4 | Forward  Reverse | TGGCCTCAGGCTTCCACTATG  CCGTTGCTTCTCTCTCTTGGG | 233 |
| *Rora* | Mouse | NM_013646 | Forward  Reverse | GTGGAGACAAATCGTCAGGAAT  TGGTCCGATCAATCAAACAGTTC | 135 |
| *Per2* | Mouse | NM_011066 | Forward  Reverse | GAAAGCTGTCACCACCATAGAA  AACTCGCACTTCCTTTTCAGG | 186 |

**Table S2. List of antibodies**

| Protein | Assay | Cat. No. | Company | Origin | Dilution | Incubation Period |
| --- | --- | --- | --- | --- | --- | --- |
| β-actin | WB | SC-47778 | Santa Cruz | Mouse | 1:3000 | Overnight, 4°C |
| CD31 | WB | ab28364 | Abcam | Rabbit | 1:500 | Overnight, 4°C |
| CYP11A1 | WB | SC-18043 | Santa Cruz | Goat | 1:200 | Overnight, 4°C |
| CYP19A1 | WB | ab18995 | Abcam | Rabbit | 1:500 | Overnight, 4°C |
| DBP | WB | 12662-1-AP | Proteintech | Rabbit | 1:500 | Overnight, 4°C |
| E4BP4 | WB | #14312 | Cell Signaling | Rabbit | 1:500 | Overnight, 4°C |
| ERα | WB | SC-8002 | Santa Cruz | Mouse | 1:200 | Overnight, 4°C |
| GAPDH | WB | SC-25778 | Santa Cruz | Rabbit | 1:3000 | Overnight, 4°C |
| PCNA | WB | CBL407 | Chemicon | Mouse | 1:500 | Overnight, 4°C |
| REV-ERBα | WB | #13418 | Cell Signaling | Rabbit | 1:500 | Overnight, 4°C |
| RORA | WB | 10616-1-AP | Proteintech | Rabbit | 1:500 | Overnight, 4°C |
| StAR | WB | SC-166821 | Santa Cruz | Mouse | 1:100 | Overnight, 4°C |
| VEGF-A | WB | ab183100 | Abcam | Rabbit | 1:400 | Overnight, 4°C |
| E4BP4 | ICC | SC-74415 | Santa Cruz | Mouse | 1:100 | Overnight, 4°C |
| Ki67 | ICC | ab15580 | Abcam | Rabbit | 1:200 | Overnight, 4°C |
| REV-ERBα | ICC | SC-100910 | Santa Cruz | Mouse | 1:100 | Overnight, 4°C |
| CD31 | IHC | ab28364 | BD Pharmingen | Rabbit | 1:200 | Overnight, 4°C |
| Cleaved caspase-3 | IHC/ICC | #9661 | Cell Signaling | Rabbit | 1:200 | Overnight, 4°C |
| CYP19A1 | IHC | ab18995 | Abcam | Rabbit | 1:100 | Overnight, 4°C |
| PCNA | IHC | CBL407 | Chemicon | Mouse | 1:200 | Overnight, 4°C |
| PER2 | IHC | Ab227727 | Abcam | Rabbit | 1:100 | Overnight, 4°C |
| REV-ERBα | IHC | 14506-1-AP | Proteintech | Rabbit | 1:100 | Overnight, 4°C |
| RORA | IHC | 10616-1-AP | Proteintech | Rabbit | 1:100 | Overnight, 4°C |
| VEGF-A | IHC | ab183100 | Abcam | Rabbit | 1:100 | Overnight, 4°C |

**Table S3. qPCR miRNA-specific stem loop-RT primers.**

| **miRNA** | **Accession** | **Forward primer** | **Universal reverse primer** |
| --- | --- | --- | --- |
| has-let-7a-5p | MIMAT0000062 | GGGTGAGGTAGTAGGTTGT | GTGCAGGGTCCGAGGT |
| hsa-let-7b-5p | MIMAT0000063 | TGTGTTGTGAGGTAGTAGGTTGT | GTGCAGGGTCCGAGGT |
| has-miR-16-5p | MIMAT0000069 | GTTTGGTAGCAGCACGTAAATA | GTGCAGGGTCCGAGGT |
| has-miR-24-3p | MIMAT0000080 | GTTTGGCTCAGTTCAGCAG | GTGCAGGGTCCGAGGT |
| has-miR-29a-3p | MIMAT0000086 | GTTTGGTAGCACCATCTGAAAT | GTGCAGGGTCCGAGGT |
| hsa-miR-191-5p | MIMAT0000440 | GGCAACGGAATCCCAAAAG | GTGCAGGGTCCGAGGT |
| has-miR-199a-5p | MIMAT0000231 | GTCCCAGTGTTCAGACTAC | GTGCAGGGTCCGAGGT |
| hsa-miR-199b-5p | MIMAT0000263 | GTTTGCCCAGTGTTTAGACTAT | GTGCAGGGTCCGAGGT |
| U6 | ENSG00000206625 | CTCGCTTCGGCAGCACA | AACGCTTCACGAATTTGCGT |
