# Supplementary material for: Secretome from estrogen-responding human placenta-derived mesenchymal stem cells rescues ovarian function and circadian rhythm in mice with cyclophosphamide-induced primary ovarian insufficiency
Source: J Biomed Sci. 2024 Oct 11;31:95. doi: 10.1186/s12929-024-01085-8 (PMC11468397; doi:10.1186/s12929-024-01085-8)

ESR1 atlas-Adipose tissue

https://www.proteinatlas.org/ENSG00000091831-ESR1/single+cell+type/adipose+tissue

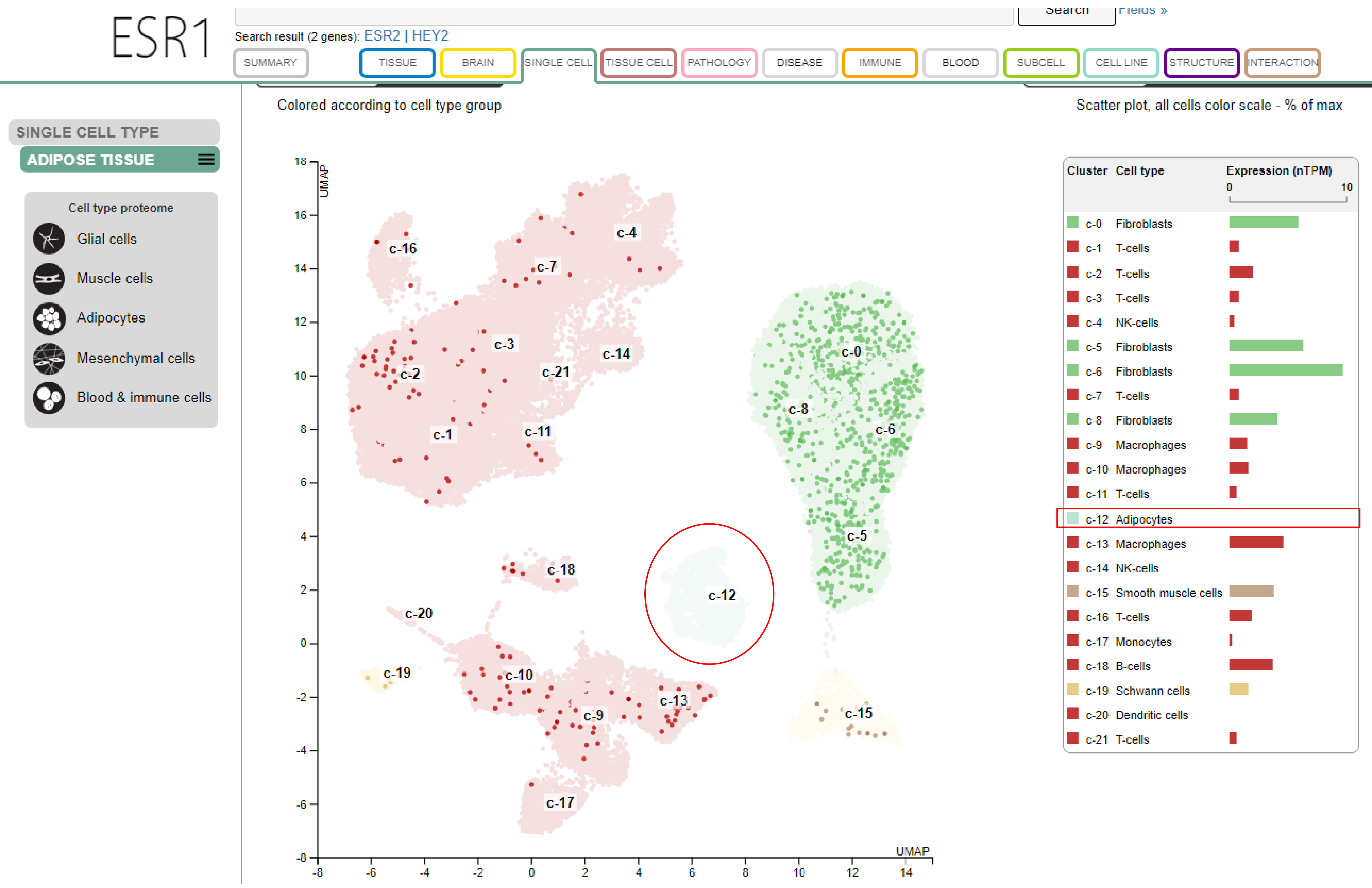

https://www.proteinatlas.org/ENSG00000091831-ESR1/tissue/adipose+tissue

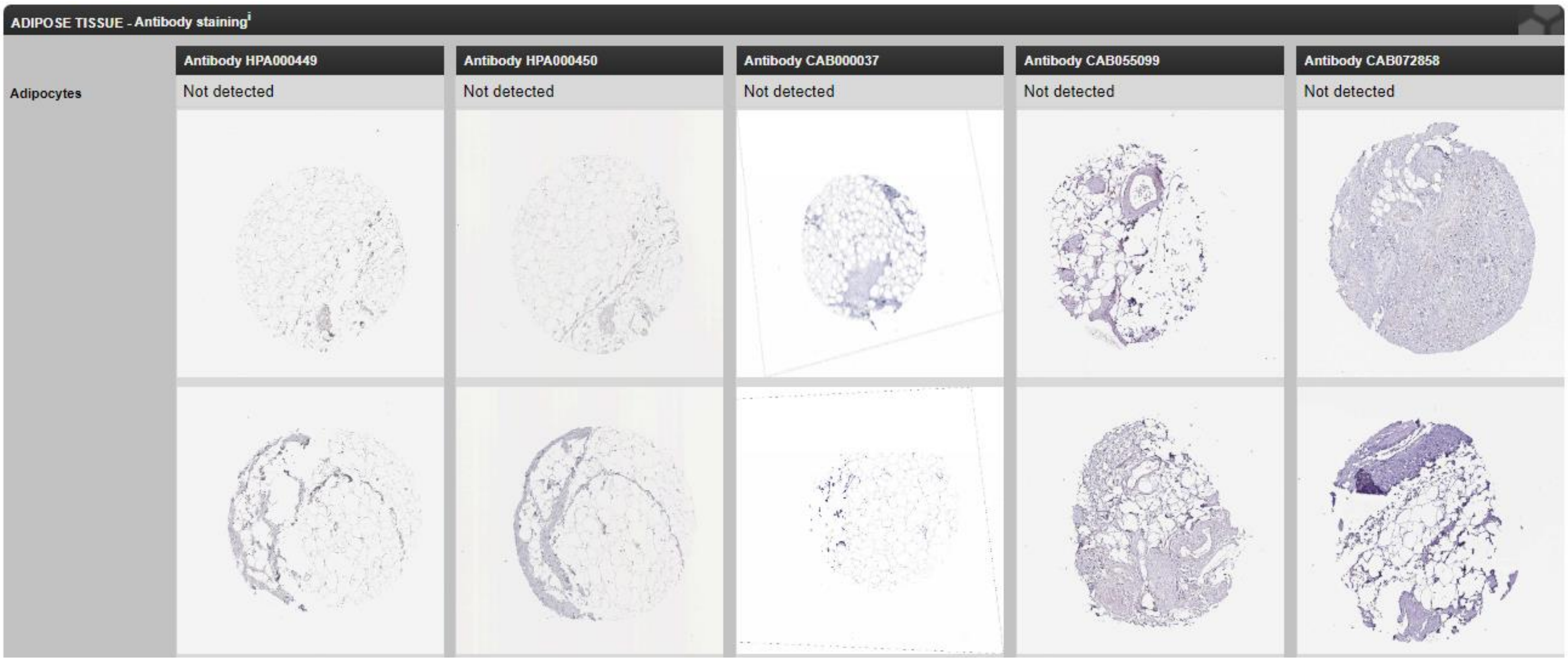

ESR1 atlas-Adipose tissue

https://www.proteinatlas.org/ENSG00000091831-ESR1/tissue+cell+type

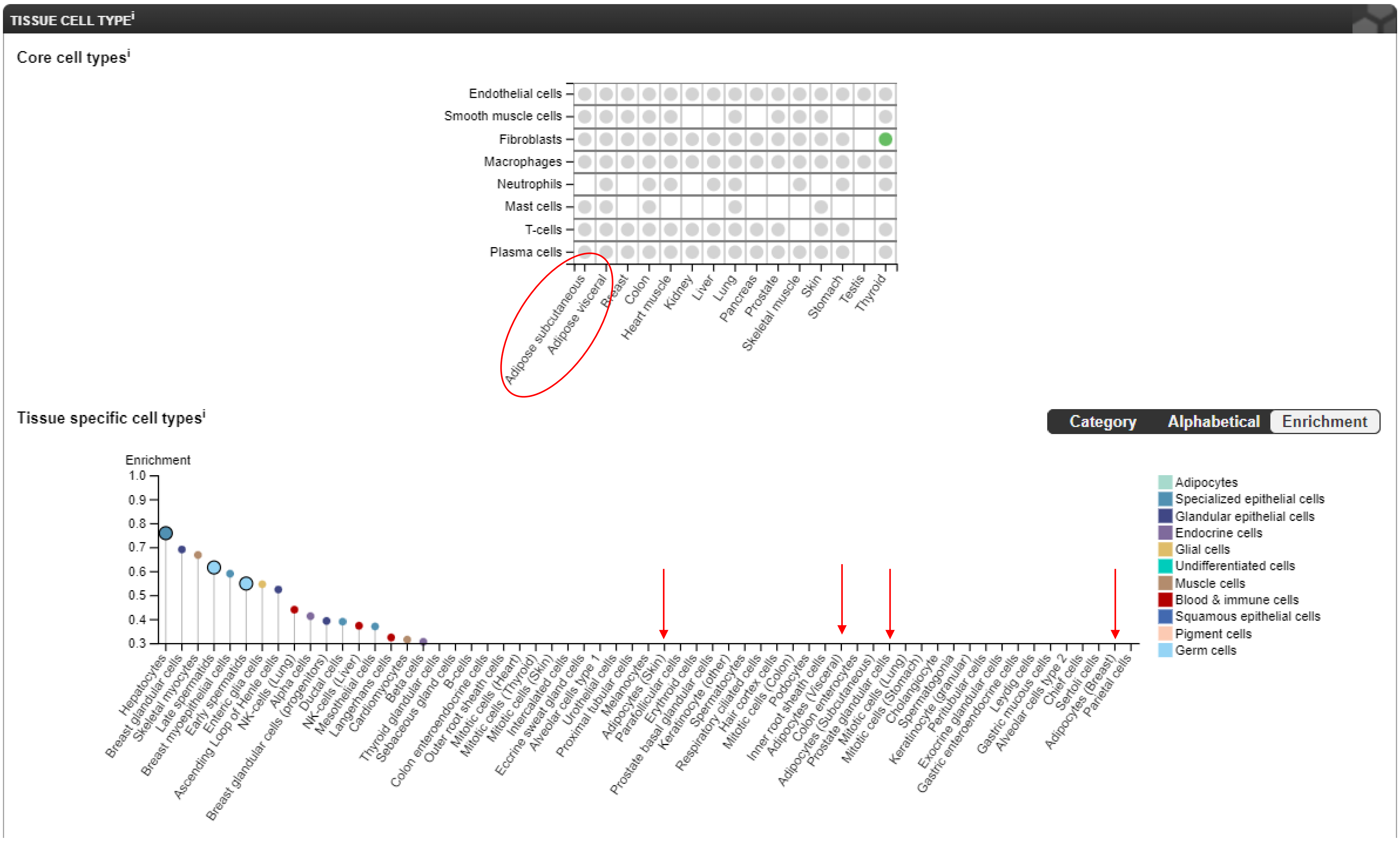

https://www.proteinatlas.org/ENSG00000091831-ESR1/tissue

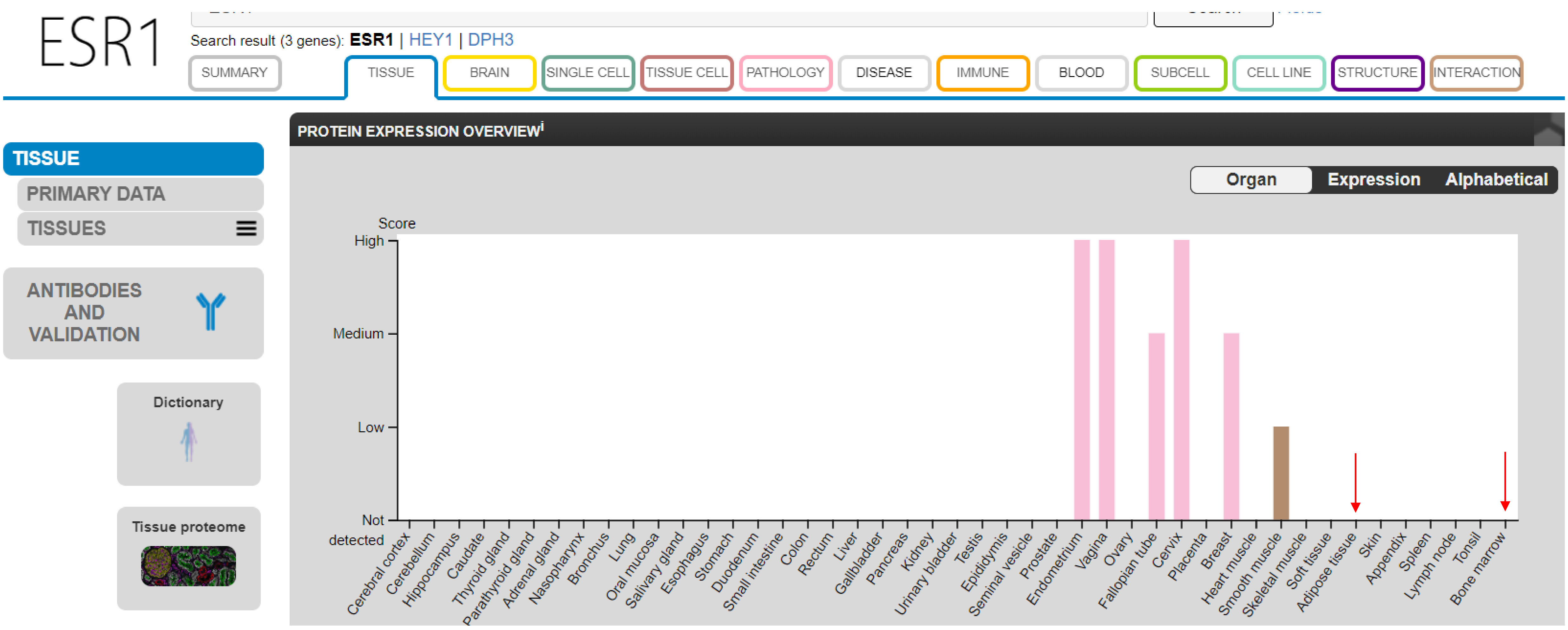

ESR1 atlas-Bone Marrow

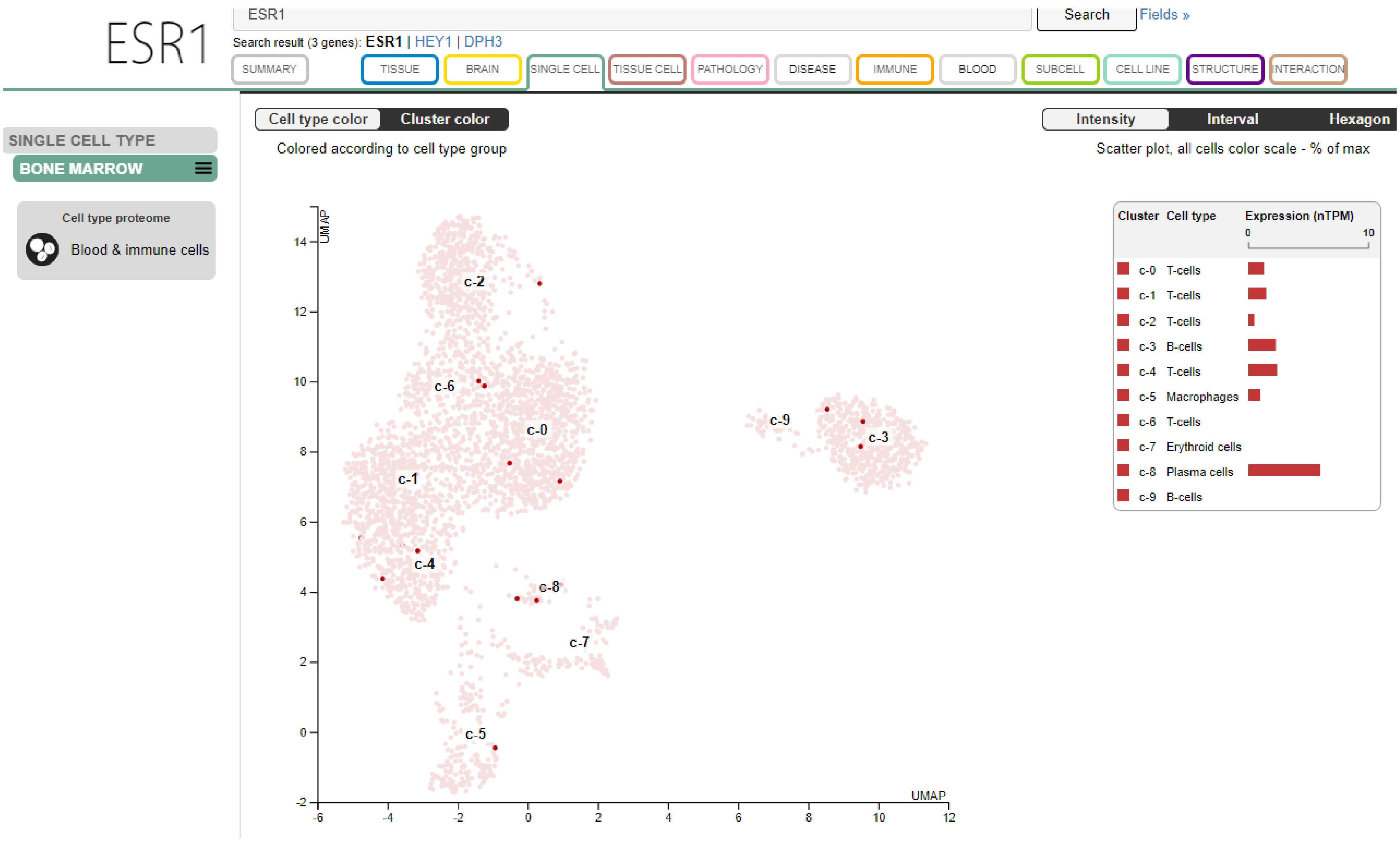

https://www.proteinatlas.org/ENSG00000091831-ESR1/tissue/bone+marrow

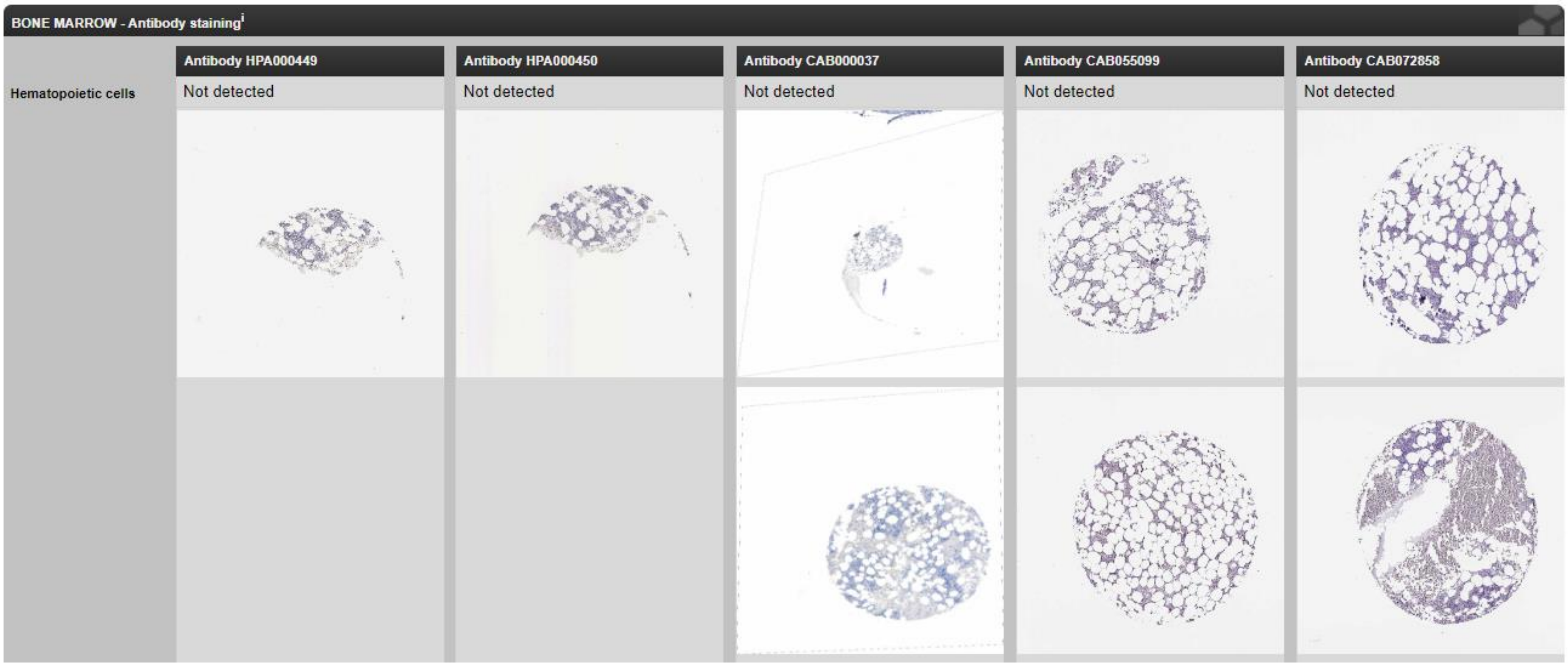

BM-MSC

https://www.nature.com/articles/s41467-024-48255-8/figures/3

a Protein expression levels of ESRRA were evaluated in BMSCs upon adipogenic induction for indicated days, comparing *Esrra*$^{fl/fl}$ mice (blue font) with *Esrra*^{AKO} mice (red font).

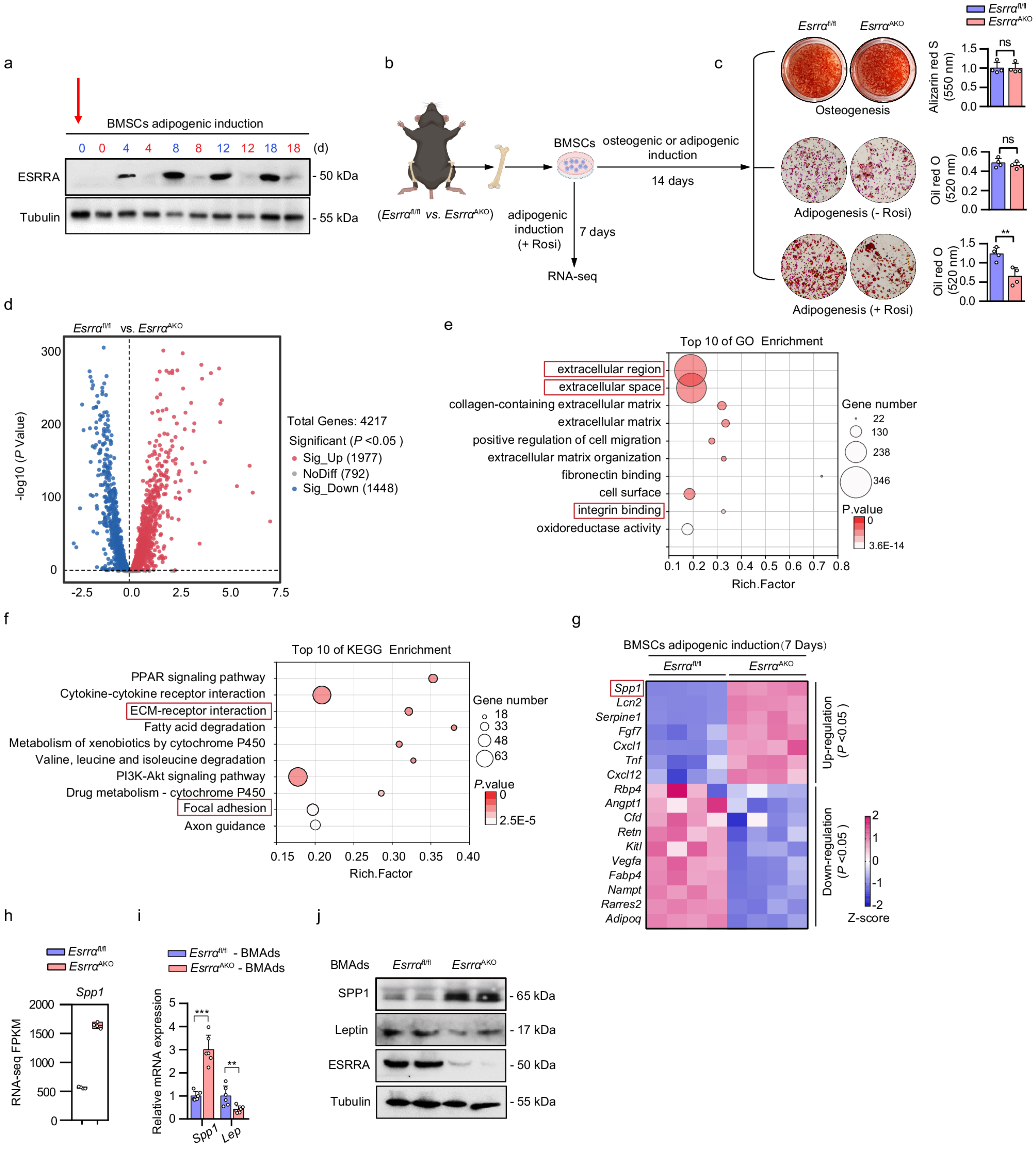

Human Adipose-derived stromal cells (ASCs)

https://www.liebertpub.com/doi/pdfplus/10.1089/ten.2006.0317

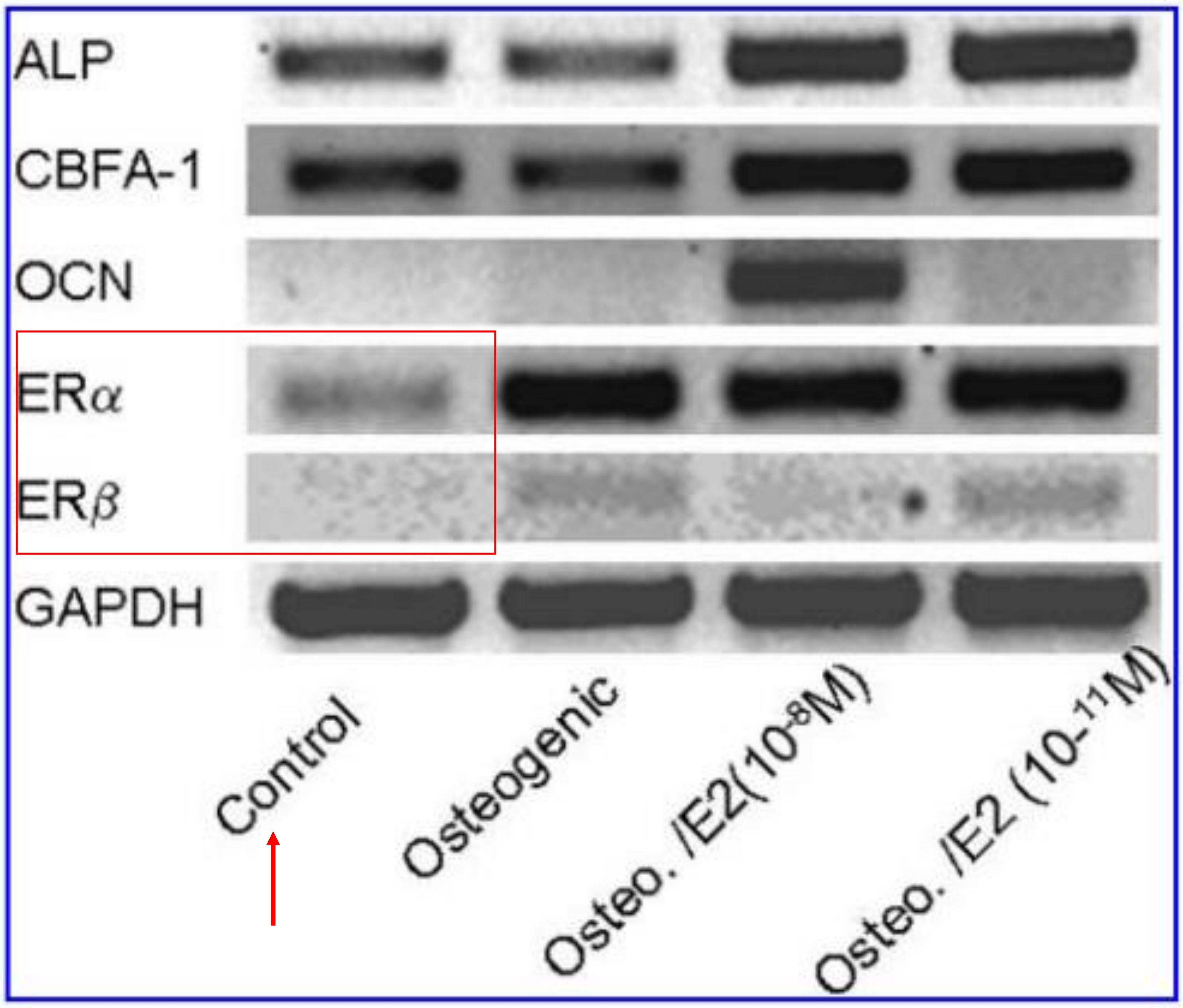

FIG. 2. RT-PCR results of osteogenic differentiation of human ASCs modulated by 17β estradiol after exposure to 7 days of differentiation medium.

UC-MSC

https://www.sciencedirect.com/science/article/pii/S0891584918313601

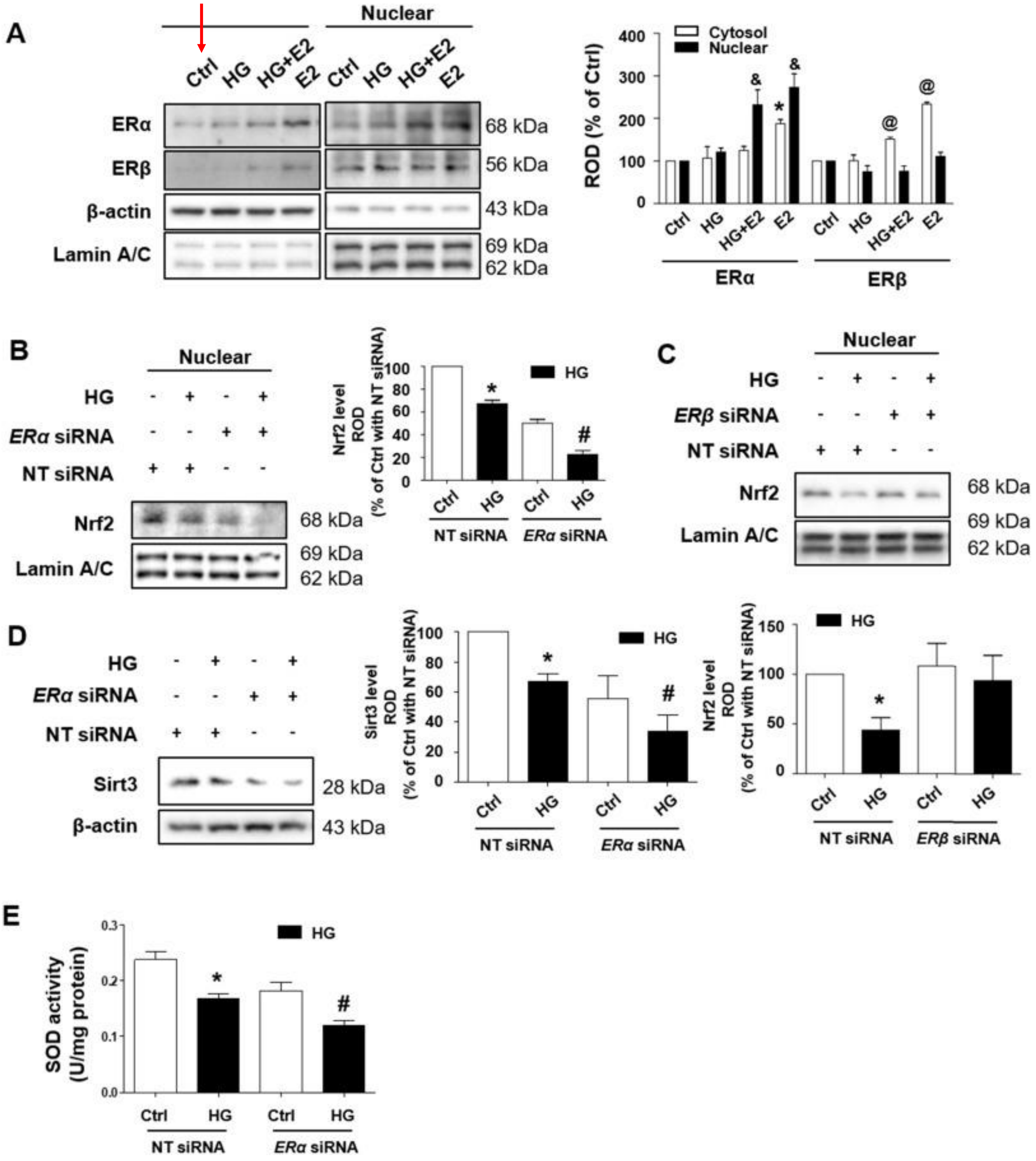

Supplement: Supplementary file 10 — Supplementary Material 10. [file 12929_2024_1085_MOESM10_ESM.pdf]
